# Understanding phylogenetic incongruence: lessons from phyllostomid bats

**DOI:** 10.1111/j.1469-185X.2012.00240.x

**Published:** 2012-08-14

**Authors:** Liliana M Dávalos, Andrea L Cirranello, Jonathan H Geisler, Nancy B Simmons

**Affiliations:** 1 Department of Ecology and Evolution, and Consortium for Inter-Disciplinary Environmental Research, State University of New York at Stony BrookStony Brook, NY 11794, USA; 2 Division of Vertebrate Zoology (Mammalogy), American Museum of Natural HistoryNew York, NY 10024, USA; 3 Department of Anatomical Sciences, State University of New York at Stony BrookStony Brook, NY 11794, USA; 4 Department of Anatomy, New York College of Osteopathic MedicineOld Westbury, NY 11568, USA

**Keywords:** adaptive convergence, incongruence, gene trees, partitioned likelihood support, phylogeny, Phyllostomidae, saturation, species trees

## Abstract

All characters and trait systems in an organism share a common evolutionary history that can be estimated using phylogenetic methods. However, differential rates of change and the evolutionary mechanisms driving those rates result in pervasive phylogenetic conflict. These drivers need to be uncovered because mismatches between evolutionary processes and phylogenetic models can lead to high confidence in incorrect hypotheses. Incongruence between phylogenies derived from morphological *versus* molecular analyses, and between trees based on different subsets of molecular sequences has become pervasive as datasets have expanded rapidly in both characters and species. For more than a decade, evolutionary relationships among members of the New World bat family Phyllostomidae inferred from morphological and molecular data have been in conflict. Here, we develop and apply methods to minimize systematic biases, uncover the biological mechanisms underlying phylogenetic conflict, and outline data requirements for future phylogenomic and morphological data collection. We introduce new morphological data for phyllostomids and outgroups and expand previous molecular analyses to eliminate methodological sources of phylogenetic conflict such as taxonomic sampling, sparse character sampling, or use of different algorithms to estimate the phylogeny. We also evaluate the impact of biological sources of conflict: saturation in morphological changes and molecular substitutions, and other processes that result in incongruent trees, including convergent morphological and molecular evolution. Methodological sources of incongruence play some role in generating phylogenetic conflict, and are relatively easy to eliminate by matching taxa, collecting more characters, and applying the same algorithms to optimize phylogeny. The evolutionary patterns uncovered are consistent with multiple biological sources of conflict, including saturation in morphological and molecular changes, adaptive morphological convergence among nectar-feeding lineages, and incongruent gene trees. Applying methods to account for nucleotide sequence saturation reduces, but does not completely eliminate, phylogenetic conflict. We ruled out paralogy, lateral gene transfer, and poor taxon sampling and outgroup choices among the processes leading to incongruent gene trees in phyllostomid bats. Uncovering and countering the possible effects of introgression and lineage sorting of ancestral polymorphism on gene trees will require great leaps in genomic and allelic sequencing in this species-rich mammalian family. We also found evidence for adaptive molecular evolution leading to convergence in mitochondrial proteins among nectar-feeding lineages. In conclusion, the biological processes that generate phylogenetic conflict are ubiquitous, and overcoming incongruence requires better models and more data than have been collected even in well-studied organisms such as phyllostomid bats.

## I. INTRODUCTION

The central premise of phylogenetics is that there is a hierarchical pattern of relationships among organisms that may be inferred by observing and analyzing homologous characters shaped by evolutionary history. A character state in two species is homologous when it is inherited from their common ancestor without modification; however, applying this definition requires an underlying species phylogeny, which itself is a hypothesis and is usually unknown. Congruence among characters is the key test of homology (Patterson, 1988), and the foundation of all phylogenetic analyses (Rieppel & Kearney, 2002). Although most characters in an organism share a common evolutionary history, differential rates of change and evolutionary mechanisms driving those rates produce incongruent phylogenies (Bull *et al.*, 1993). Incongruence among phylogenies estimated from different sets of characters is pervasive (Rokas *et al.*, 2003). Phylogenetic conflict has become a more acute problem with the advent of genome-scale data sets. These large data sets have confirmed that phylogenetic conflict is common, and frequently the norm rather than the exception (Waddell *et al.*, 1999; Leebens-Mack *et al.*, 2005; Jeffroy *et al.*, 2006; Rodríguez-Ezpeleta *et al.*, 2007).

Early efforts to understand phylogenetic incongruence revealed that taxonomic sampling (Graybeal, 1998), the number of characters sampled (Rosenberg *et al.*, 2002), and methods of analyses (Felsenstein, 1978) can all affect estimates of phylogeny. Large data sets have helped establish that high rates of change leading to saturation (Phillips, Delsuc & Penny, 2004; Dávalos & Perkins, 2008), and biological processes leading to different gene trees are common (Bapteste *et al.*, 2005; Degnan & Rosenberg, 2006). In-depth analyses of specific genes in the context of multi-locus phylogenies have also shown that adaptive evolution leading to convergence, once thought to be extremely rare (Patterson, 1988), is as much a source of conflict among gene trees as it is between morphological and molecular phylogenies (Delsuc *et al.*, 2001; Reiss, 2001; Ruedi & Mayer, 2001; Castoe *et al.*, 2009; Li *et al.*, 2010; Liu *et al.*, 2010).

Despite these advances in understanding phylogenetic conflict, few studies systematically analyze conflict with empirical data sets (e.g. Daubin, Moran & Ochman, 2003; Rokas *et al.*, 2003; Canback, Tamas & Andersson, 2004; Dávalos & Perkins, 2008), fewer extend to morphology (e.g. Delsuc *et al.*, 2006; Gaubert *et al.*, 2005), and very few compare morphological phylogenies, rather than hypotheses based on traditional systematics (e.g. Ragsdale & Baldwin, 2010). The dearth of analyses focusing simultaneously on morphology and molecular data is troubling because morphological data are the only characters available for much of the Tree of Life, and their use is indispensable (Wiens, 2005, 2009). Morphological expertise and data are in short supply relative to their molecular counterparts (de Carvalho *et al.*, 2008), preventing in-depth analyses of methodological and biological drivers of conflict.

**Figure 1:**
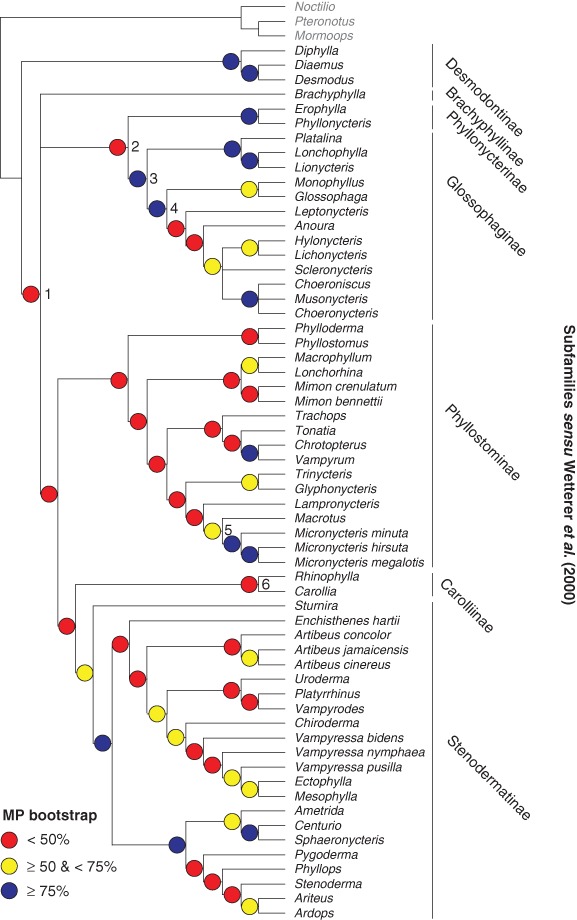
Strict consensus of maximum parsimony (MP) trees and summary of bootstrap support values from Wetterer *et al.* (2000). Numbered nodes show resolutions conflicting with both analyses of expanded morphological data or with molecular data. Following Simmons (2005)*Vampyressa nymphaea* here = *Metavampyressa nymphaea* in subsequent figures. Classification follows Wetterer *et al.* (2000), outgroups are shown in grey type.

**Figure 2:**
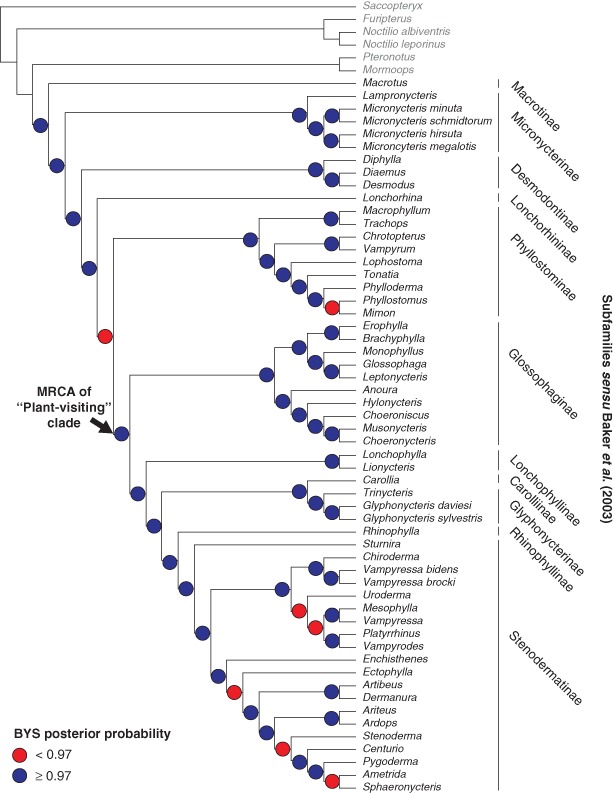
Fifty percent majority-rule consensus of Bayesian (BYS) trees and posterior probabilities from Baker *et al.* (2003). Classification follows Baker *et al.* (2003), outgroups are shown in grey type. MRCA, most recent common ancestor.

Herein, we extend a preexisting morphological data set for the family Phyllostomidae (Mammalia: Chiroptera) with the goal of systematically evaluating sources of phylogenetic conflict. Phyllostomids are an ideal system in which to investigate conflict because early morphological (Fig. 1) and multi-locus molecular hypotheses (Fig. 2) are clearly incongruent (Wetterer, Rockman & Simmons, 2000; Baker *et al.*, 2003). Subsequent analyses of large molecular data sets have confirmed significant conflict by corroborating aspects of the phylogeny of Baker *et al.* (2003) that conflict directly with morphological analyses (Datzmann, von Helversen & Mayer, 2010). For this reason, and because it is the most taxonomically comprehensive molecular phylogeny, we use the phylogeny of Baker *et al.* (2003), hereafter called the “reference phylogeny”, as the basis for comparisons with results of morphological, molecular, and combined data.

Phyllostomids are also ideal for investigating the forces underlying phylogenetic conflict because their ecology is relatively well understood (Santana & Dumont, 2009; Dumont *et al.*, 2012), enabling tests of adaptive convergence as a source of conflict. Our study has three objectives: (*i*) to match taxonomic sampling in morphological data to the molecular analyses; (*ii*) to control for methodological sources of incongruence by estimating phylogenies using models of character evolution with both molecular and morphological data; and (*iii*) to analyze the biological drivers of phylogenetic conflict across multiple data sets. Our analyses uncovered multiple biological processes operating on both morphological and molecular data sets that result in systematic biases and conflicting estimates of phylogeny. The results can help guide both phylogenetic and evolutionary data collection in this ecologically diverse mammalian family.

## II. MATERIALS AND METHODS

### (1) Data collection

#### (a) Taxonomic sampling, outgroups, and tree rooting

Our taxonomic sample included all currently recognized phyllostomid genera with the exceptions of *Neonycteris* and *Xeronycteris*, known only from their holotypes and paratypes, for a total of 71 ingroup species. We sampled multiple species of *Artibeus*, *Micronycteris*, *Vampyressa* and *Mimon* because monophyly of these genera has been questioned (Owen, 1991; Lee, Hoofer & Van Den Bussche, 2002; Baker *et al.*, 2003, 2000; Hoofer & Baker, 2006; Tavares, 2008). In some cases, we selected two species per genus to facilitate inclusion of both morphological and molecular data (e.g. *Mystacina*, *Pteronotus*, *Phyllonycteris*, *Anoura*, *Lonchophylla*, *Glyphonycteris*, *Lophostoma*, *Carollia*, and *Artibeus*, subgenus *Artibeus*). Representatives of five other noctilionoid families (Mormoopidae, Noctilionidae, Mystacinidae, Furipteridae and Thyropteridae) *sensu*Teeling *et al.* (2005) were included in our study as outgroups. To polarize character states, we rooted trees with *Saccopteryx bilineata*, a member of the family Emballonuridae. A total of 62 genera, 56 of them phyllostomids, were sampled. For ease of discussion, and unless otherwise noted, we used the family-level classification proposed in the reference phylogeny (Fig. 2).

#### (b) Morphological data

The morphological data set presented here is an enhanced version of the Wetterer *et al.* (2000) data set. We improved on those data by scoring taxa at the species level, adding species to provide a better match with the taxonomic sample from the molecular data, and adding characters from additional sources (e.g. Simmons & Conway, 2001). The revised data comprise 220 morphological characters scored in 80 species (2.75 characters/taxon), adding 82 new characters to the original matrix.

We used reductive character coding, splitting logically independent features into separate characters that we coded hierarchically *sensu*Simmons (1993) and Wilkinson (1995). In hierarchical coding a ‘mother’ character codes for, as an example, the presence of a structure. ‘Daughter’ characters might then code for shape, size, or colour of the feature, but are only coded for taxa in which the feature is present. If the feature is absent, the taxon is scored as inapplicable: ‘-.’ We occasionally fused an independent and dependent character when there was only a single dependent character in the matrix. We used “any instance” coding for polymorphic characters (fixation of the primitive state = 0; polymorphism or fixation of the apomorphic state = 1) to accommodate situations in which variants were rare, characters were binary, and some assessment of the derived condition could be made *a priori* (e.g. by comparison with outgroups Wiens, 2000). The remaining instances of intra-specific polymorphism were scored using polymorphic coding (0/1; Wiens, 2000). Only five morphological characters (2% of our morphological matrix) were coded as polymorphic. Taxa had 6.8–67.3% missing data (mean = 28.6% missing; median = 66.5 characters per taxon). Most of the morphological characters were binary (152 = 69%), and the remaining characters were multistate (68 = 31%). Forty-four multistate characters described progressive gradations in size, shape, degree of development, meristic counts, or colour patterns, and these were treated as ordered in analyses. We used MacClade v.4.08 to order and map characters and to define character groups, character partitions, and taxon sets (Maddison & Maddison, 2003).

#### (c) Molecular data

We obtained sequences of species exemplars from GenBank for 12S and 16S mitochondrial ribosomal sequences (mtrDNA), complete mitochondrial cytochrome *b* (*CYTB*), partial mitochondrial cytochrome oxidase I (*COX1*), and a 1.3 kb fragment of the nuclear recombination activating gene 2 (*RAG2*) (Van Den Bussche, Hudgeons & Baker, 1998; Wright *et al.*, 1999; Hoffmann & Baker, 2001; Baker *et al.*, 2003, 2000; Dávalos & Jansa, 2004; Porter & Baker, 2004; Clare *et al.*, 2007; Dávalos, 2007; Porter *et al.*, 2007). Three ingroup species (*Lichonycteris obscura*, *Phyllonycteris poeyi*, *Scleronycteris ega*) and one outgroup (*Mystacina robusta*) had no sequence data, and were excluded from molecular-only analyses. Because sampling depended on the availability of sequences published in GenBank, roughly 23% of the molecular matrix was missing data. Supermatrix analyses with >90% missing data are viable for estimating phylogenies (Smith, Beaulieu & Donoghue, 2009), although the proportion of missing data herein is higher than generally found in targeted sequencing studies. Of sequences available from 76 species, 24% were missing from mtrDNA, 9% from *CYTB*, 38% from *COX1*, and 10% from *RAG2*. The amount of missing data did not differ significantly among subfamilies and outgroups sampled (one-way ANOVA: F_obs_ = 1.385; *P* = 0.202). Species names and GenBank accessions of sequences are shown in online Appendix S1.

### (2) Measuring character state exhaustion or saturation

#### (a) Morphological data

Morphological data sets can exhaust character states as they incorporate more species so that further changes erode phylogenetic signal (Wagner, 2000). Instead of accumulating new character states as new lineages arise, the number of character states appears to be constrained in most empirical data sets (Wagner, 2000). Constraints on design arising from development or function can thus result in homoplasy, as species end up sharing character states that are not inherited from their most recent common ancestor (Sanderson & Donoghue, 1989; Wake, 1991; Donoghue & Ree, 2000; Masters, 2007). This leads to long-branch attraction, an artifact of these constraints on character states that has been well studied with molecular data (Huelsenbeck, 1997; Graybeal, 1998), but that is seldom tested with morphological data.

We evaluated saturation or exhaustion in character states in the morphological data using the approach of Wagner (2000). First, we converted all ordered characters to unordered, then optimized these on the maximum parsimony phylogeny using ACCTRAN (accelerated transformation), and counted the number of states and steps for each branch, beginning with the oldest branches in the phylogeny based on the dated phylogeny of Dumont *et al.* (2012). Second, we identified the portion of the state : step curve where states were added linearly as a function of steps by fitting a segmented regression model to the data with a single breakpoint (Muggeo, 2008). All hypotheses about character-state acquisition—explained below—predict a similar linear relationship at the base of the tree, so this linear part of the curve was excluded in subsequent analyses. Finally, the non-linear remainder of the data was used to test the null hypothesis of consistent addition of states, by categorizing steps into two categories: those that added new states and those that did not. If the morphological data were not saturated, adding or not adding new states would be randomly distributed along the step accumulation curve, i.e. each step would have equal probability of being in either category. We tested this null hypothesis of consistent addition of new states using a Mann-Whitney test (Mann & Whitney, 1947) to compare the ranks of the number of steps in the two categories.

We also examined the fit of the state:step data to two alternative hypotheses about character-state acquisition: (*i*) a rarefaction model of character state saturation, expected if there was a ceiling in the number of character states; and (*ii*) a power function relating new states to steps, expected if only taxa possessing extreme morphology give rise to descendants with derived character states. Based on Wagner (2000), the rarefaction model or finite-state hypothesis was fitted as:

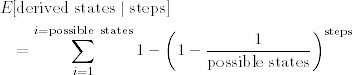


The power function or ordered-states hypothesis was fitted in relation to the constants *b* and *c*, as:

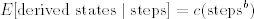


All statistical analyses were conducted in R (R Development Core Team, 2010) and curves were fitted to the data using the *nls* function of R core. We compared the resulting models using the Akaike information criterion (AIC) scores. The AIC measures goodness-of-fit between the observations and the fitted model, with lower scores indicating better fits. The best-fit model has the lowest AIC, and other models were compared to it by calculating the difference in AIC relative to the best model, or Δ_AIC_. We applied the criteria of Burnham & Anderson (2002), whereby a Δ_AIC_ < 2 indicates substantial support, Δ_AIC_ of 4–7 indicates considerably less support, and a Δ_AIC_ > 10 indicates no empirical support for the model in question.

#### (c) Molecular data and base composition bias

To provide a measure of saturation comparable across data sets, we quantified the slope of uncorrected against corrected distances for individual codon positions and stems and loops of the mitochondrial ribosomal genes. Corrected distances were calculated using PAUP* v.4.0b10 (Swofford, 2002) by applying the best-fit maximum likelihood (ML) model selected using MrAIC.pl v.1.4.3 (Nylander, 2004) for individual genes. We used PAUP* to quantify pairwise uncorrected and corrected distances, and the *slope* function of Microsoft Excel v.12.2.8 to calculate the slopes of the curves.

Three strategies were applied to reduce the impact of saturation on phylogeny estimation and tree comparisons. First, we applied partitioned models in maximum likelihood (ML) and Bayesian (BYS) analyses to minimize bias from significant changes in base composition superimposed on saturated sequences without imposing onerous time penalties on phylogeny searches (Dávalos & Perkins, 2008). Second, we applied codon and partitioned models in likelihood-based tree comparisons (see Section II.5). Third, saturated positions were excluded/down-weighted to decrease their influence on phylogeny estimation.

Base composition changes can provide strong ahistorical signals that, when superimposed on saturated data, produce strongly supported incorrect phylogenies (Dávalos & Perkins, 2008). We investigated base compositional bias using the *χ*^2^ test implemented in PAUP* for the entire alignment, for protein-coding genes at individual codon positions, and for stems and loops for the mtrDNA.

### (3) Inferring phylogenies

We conducted phylogenetic analyses using: (*i*) morphological characters only (80 species × 220 characters), (*ii*) molecular characters only (76 species × 6032 characters), and (*iii*) combined morphological and molecular data (80 species × 6252 characters).

#### (a) Molecular data partitions and alignment

We partitioned the data into loci and/or sites within loci to reflect expected differences in rates of molecular evolution (Table 1). We used functional and structural approaches to ensure that the alignments maximized sequence homology, thereby providing the best estimate of phylogeny for each gene. We used transAlign v.1.2 (Bininda-Emonds, 2005) to align protein-coding sequences (*CYTB*, *COX1*, and *RAG2*) by translating nucleotides to amino acids, aligning with clustalw v.1.83 with default settings (gap opening of 10, gap extension penalty of 0.2) (Thompson, Higgins & Gibson, 1994), and back-translating to nucleotides.

**Table 1:** Data partitions for molecular data applied in each partitioning scheme

Name	Data partitions	No. of RAxML parameters	Log-likelihood
1P	All data	9	−90681
2a	mtr, coding	18	−90511
2b	mt, *RAG2*	18	−89996
3a	mtr, mt coding, *RAG2*	27	−89828
3b	mtr, pos 1 + 2, 3	27	−89323
4	mtr, pos 1, 2, 3	36	−89168
5	mtr, mt pos 1 + 2, 3; *RAG2* pos 1 + 2, 3	45	−88094
6a	mtr 12S, 16S; mt pos 1 + 2, 3; *RAG2* pos 1 + 2, 3	54	−89132
6b	mtr 12S, tRNA^val^, 16S; pos 1, 2, 3	54	−88067
6c	mtr stems, loops; mt pos 1 + 2, 3; *RAG2* pos 1 + 2, 3	54	−87370
7a	mtr, mt pos 1, 2, 3; *RAG2* pos 1, 2, 3	63	−87952
7b	mtr stems, loops, tRNA^val^; mt pos 1 + 2, 3; *RAG2* pos 1 + 2, 3	63	−87383
8a	mtr 12S, 16S; mt pos 1 + 2, 3; *RAG2* pos 1 + 2, 3	72	−87925
8b	mtr stems, loops; mt pos 1, 2, 3; *RAG2* pos 1, 2, 3	72	−87228
9a	mtr 12S, tRNA^val^, 16S; mt pos 1, 2, 3; *RAG2* pos 1, 2, 3	81	−87917
9b	mtr stems, loops, tRNA^val^; mt pos 1, 2, 3; *RAG2* pos 1, 2, 3	81	−87241
9c	mtr 12S stems, loops, tRNA^val^, 16S stems, loops; mt pos 1 + 2, 3; *RAG2* pos 1 + 2, 3	81	−87344
11	mtr 12S stems, loops, tRNA^val^, 16S stems, loops; mt pos 1, 2, 3; *RAG2* pos 1, 2, 3	99	−87202

1P, one partition; mt, mitochondrial; mtr, mitochondrial ribosomal partition; pos, positions; *RAG2*, recombination activating gene 2; RAxML, rapid algorithm for maximum likelihood; tRNA^val^, transfer RNA valine.

We aligned the mitochondrial loci encoding ribosomal 12S, tRNA^val^, and 16S (mtrDNA) individually using MAFFT v.6.611b (Katoh & Toh, 2008*b*). Based on benchmark rRNA analyses (Katoh & Toh, 2008*a*), we applied the Q-INS-i algorithm. The resulting mtrDNA alignment was 2837 base pairs long, with gaps covering <9% of any one sequence. We then inferred the secondary structure of the mitochondrial ribosomal loci using the *Artibeus jamaicensis* sequence (Pumo *et al.*, 1998) and the proposed secondary structure for mammals (Springer & Douzery, 1996; Burk, Douzery & Springer, 2002). Jalview (Clamp *et al.*, 2004) was then used to profile-align the proposed secondary structural sequences for mammals with the alignment initially generated for our data set. We then confirmed the definition of the stem and loop regions, adjusting these by eye when necessary.

#### (b) Model selection for molecular data

We used MrAIC and the Phyml v.2.4.4 algorithm (Guindon & Gascuel, 2003) to identify the best model of sequence evolution for each partition. MrAIC calculates the AIC and the second-order AIC (AIC*c*). The AIC*c* more strongly penalizes the addition of model parameters, and was appropriate for these data, as the ratio of nucleotides to number of parameters was <40 (Posada & Buckley, 2004). All models were from the general time reversible (GTR) family of molecular evolution models (Tavaré, 1986), with or without four discrete rate categories approximating a gamma (Γ) distribution of rate variation across sites (GTR+Γ) (Yang, 1996).

#### (c) Selecting a partitioning scheme for molecular data

Partitioning schemes ranged from no partitioning to separating the stems and loops of the two ribosomal mitochondrial genes, the tRNA, and each codon position in both mitochondrial and nuclear loci (Table 1). We compared partitioning schemes using the AIC*c*, the Bayes information criterion (BIC), and a decision-theoretic (DT) approach first proposed by Minin *et al.* (2003). We used the equations of McGuire *et al.* (2007) to calculate the AIC*c* and BIC, replacing the harmonic mean of log likelihoods (HMLL) with maximum likelihood values obtained from applying the GTRGAMMA algorithm under different partition schemes with a fixed topology in RAxML v.7.0.48 (Stamatakis, 2006*b*). Each search consisted of 10 separate starting points. The decision-theoretic approach also required the ML scores from each partitioning scheme, and included a penalty for over-fitting parameters, as measured by greater variance in tree branch-length estimates (Minin *et al.*, 2003). We modified the fixed-topology version of the DT-modsel script to compare the ML scores and trees obtained under the 18 different partitioning schemes (Minin *et al.*, 2003). We then applied the selected best partitioning schemes in both RAxML bootstrap and Bayesian analyses using parallel MrBayes v.3.1.2 (Ronquist & Huelsenbeck, 2003). The number of free parameters in partitioned RAxML analyses is shown in Table 1. Bayesian partitioned analyses often had fewer parameters, as MrBayes can implement simpler models than the GTR+Γ (e.g. HKY).

#### (d) Parsimony analyses

We performed parsimony analyses running the command-line Unix version of PAUP*. The heuristic search option with a random taxon addition sequence (1000 repetitions), tree-bisection-reconnection (TBR) branch swapping, and the collapse of branches with a maximum length of zero was used to identify most-parsimonious trees. Bootstrap analyses applied 10 repetitions of random taxon addition sequence in 1000 bootstrap pseudoreplicates.

#### (e) Maximum likelihood analyses

We used RAxML to conduct maximum likelihood tree searches. First, we obtained the best estimate of phylogeny by analysing all the data using the GTRGAMMA algorithm implementing the GTR+Γ model with 25 discrete rate categories. This algorithm provided comparable likelihood scores across analyses. Second, the resulting topology was fixed in ML analyses of alternative partitioning schemes to reduce computational time. Finally, ML phylogenies under the best partitioning schemes were estimated using the GTRGAMMA algorithm and bootstrap support (1000 pseudoreplicates) was calculated for these topologies using the rapid algorithm implementing the GTRCAT routine (Stamatakis, 2006*a*; Stamatakis, Hoover & Rougemont, 2008).

#### (f) Bayesian analyses

We conducted multiple sets of phylogenetic analyses using different character partitions as described above. We ran parallel MrBayes on multiple servers (see Section VI). Bayesian analyses applied the best model under the AIC*c* option of MrAIC for the whole dataset, or individual partitions. For the morphology partition, we specified the Markov k-state variable model (Mkv) with a gamma-distributed rate parameter (Mkv+Γ) (Lewis, 2001). We also used this model for the morphological data when running combined analyses, along with best-fit models for the molecular partitions. We conducted partitioned analyses with variable rates (ratepr = variable); and unlinked base frequencies, transition/transversion rate ratios, rate matrices, gamma distributions parameters between individual partitions, and sampled trees and parameters every 1000 generations from four chains (one unheated). The chains ran at least four times for 5000000 generations for morphological data (±Γ); 10000000, 15000000, or 20000000 generations for molecular partitioned and unpartitioned analyses; and 15000000 generations for partitioned and unpartitioned combined analyses. Molecular and combined data analyses began with a user-defined tree without branch lengths derived from ML searches. We determined if chains converged to a stationary distribution by examining the average standard deviation of split frequencies and comparing the log-likelihoods of chains from different runs using ANOVA to establish that post burn-in samples were indeed sampling from islands of similar log-likelihoods and could be used in subsequent tree comparisons. Non-stationary samples were discarded as burn-in.

### (4) Data and phylogeny deposition

Matrices for all data types, including regions assigned to stems and loops of mitochondrial 12S, tRNA^val^ and 16S, and summary trees in NEXUS format were deposited in TreeBASE (http://treebase.org/), a database established for documenting phylogenetic data and results under submission ID 11671 (Morell, 1993).

### (5) Measuring phylogenetic incongruence

To measure incongruence, we compared node support values across phylogenies derived from alternative combinations of data partitions and analytical methods. Nodes were considered in conflict if alternative resolutions had either ≥50% bootstrap support or ≥0.97 Bayesian posterior probability (BPP).

To investigate the relative contribution of individual partitions to phylogenetic resolution and conflict in a parsimony framework, we quantified the per-character retention index (RI) given the reference phylogeny. The RI measures the degree to which identical character states can be retained as homologies in the tree, and is insensitive to both tree length and the inclusion of unique derived states (Farris, 1989*a*). The RI ranges from 0 to 1, with high values indicating complete agreement between changes in the character and the tree, and low values indicating the character can only be interpreted as homoplasy in the tree (Farris, 1989*b*). We used the Kolmogorov-Smirnov test to compare the frequency distributions of the RI between different types of data (Massey, 1951), the R package *reldist* to visualize the differences in their relative density (Handcock & Morris, 1999), and the G-test of goodness-of-fit to compare observed numbers of characters with RI = 1 to the proportion expected if all character classes were equally represented. Following Wetterer *et al.* (2000), morphological characters were subdivided among six classes: pelage and integument, skull and dentition, postcranium, hyoid apparatus, tongue, and internal [subsuming the few characters that Wetterer *et al.* (2000) apportioned among the brain, digestive and reproductive tracts].

We used partitioned likelihood support (PLS) to locate support for particular nodes in a likelihood framework (Lee & Hugall, 2003), modified as explained below. Analyzing the PLS required using a subset of 56 taxa for which data were available for every partition (see TreeBASE submission). We implemented PLS by obtaining ML scores and optimal branch lengths and model parameters for the best tree and collapsed-node topologies using the *baseml* function in PAML v. 4.3 (Yang, 2007*b*). These model parameters (including branch lengths) were then applied in *baseml* optimizations of the topologies for each partition using the *in*.*baseml* file to enforce the same parameters across partitions. PAML outputs were parsed using custom Perl scripts available upon request from the authors. We introduced one important departure from Lee & Hugall's (2003) method: they enforced a single model across all partitions. We obtained site likelihoods with both a single model for all the molecular data (Lee & Hugall, 2003), and with partition-specific parameters, but enforcing the same branch lengths, using the G option in *baseml*. The significance of the per-partition differences was tested using the weighted Shimodaira-Hasegawa (WSH) (Shimodaira & Hasegawa, 1999) and approximately unbiased (AU) (Shimodaira, 2002) tests in consel v. 0.1 (Shimodaira & Hasegawa, 2001). One random tree was included in likelihood-based tree comparisons to ensure the range of differences between log-likelihoods was large, making assessments of significance comparable between analyses (van Rij *et al.*, 2003).

Finally, we compared the topologies derived from different analyses using site likelihoods derived from the molecular data and its partitions using *codeml* (for codons) and *baseml* (for nucleotides) functions in PAML. As in PLS analyses, the significance of the differences in likelihood scores was tested using the WSH and AU tests in consel, including one random tree.

### (6) Testing for adaptive convergence

#### (a) Morphological data

Adaptive convergent evolution is a special case of homoplasy caused by selective pressures to perform similar functions (Reiss, 2001). To identify spurious clades resulting from adaptive convergence in morphological characters, we followed the criteria proposed by Wiens, Chippindale & Hillis (2003): (*i*) evidence that the clade is incorrect (e.g. clade not recovered in the reference phylogeny); (*ii*) support for the clade based on morphology; and (*iii*) ecological relevance of the incorrect clade. We then examined unambiguous character transformations inferred along the branches defining the conflicting nodes to assess the relationship between characters underlying the node and ecological function, and develop a list of potentially convergent characters to be subsequently excluded. This tested the impact of such characters on phylogenetic analyses of morphology.

#### (b) Molecular data

We investigated adaptive convergence in molecular data by modifying the criteria of Wiens *et al.* (2003) as follows: (*i*) evidence that the clade is incorrect; (*ii*) support for the spurious clade from functionally relevant parts of the gene; (*iii*) evidence of a link between the gene and ecological function; and (*iv*) evidence of selection operating on the gene consistent with the proposed function. The first two criteria were evaluated using partitioned likelihood support, and the link between the gene and function based on published accounts of the role of the gene/gene regions. A maximum likelihood approach was used to quantify the shift in selection pressure expected to occur when a gene evolves in a convergent manner in independent lineages. This approach estimates the ratio of non-synonymous to synonymous substitutions (*K*_*a*_*/K*_*s*_) across codons and branches in the reference phylogeny. Under purifying selection, deviations from the functional phenotype are selected against, and amino acid replacements resulting from non-synonymous substitutions are rare relative to synonymous substitutions (*K*_*a*_*/K*_*s*_ < 1). Under positive selection, there is selection for a particular phenotype, and amino acid replacements become common relative to synonymous substitutions, raising the *K*_*a*_*/K*_*s*_ ratio. We used the likelihood ratio tests of Bielawski & Yang (2003, 2004) to estimate the variability of *K*_*a*_*/K*_*s*_ ratios in genes and lineages, and to locate codons where selection has shifted in a positive direction relative to the background using the Bayesian empirical Bayes approach. The test compares two models: a null nearly neutral model that assumes the alignment can be partitioned into two classes of codons, each with its own *K*_*a*_*/K*_*s*_ ratio; and a more complex model with three classes of codons, where *K*_*a*_*/K*_*s*_ < 1 (purifying selection), *K*_*a*_*/K*_*s*_ = 1 (neutral evolution), and a *K*_*a*_*/K*_*s*_ ratio that varies among foreground lineages, those of interest, and background lineages (all the rest). Analyses of models fitting shifts in selection for each lineage, and corresponding null models, ran with five different starting values of *K*_*a*_*/K*_*s*_ (0.001, 0.01, 0.1, 1, and 10) to ensure the algorithm reached the global maximum likelihood optimum, rather than a local optimum. Although proposed to test positive selection, the test can identify codons undergoing differential selection—more positive or negative—on designated branches of the phylogeny.

## III. RESULTS

### (1) Saturation

#### (a) Morphological data

**Figure 3:**
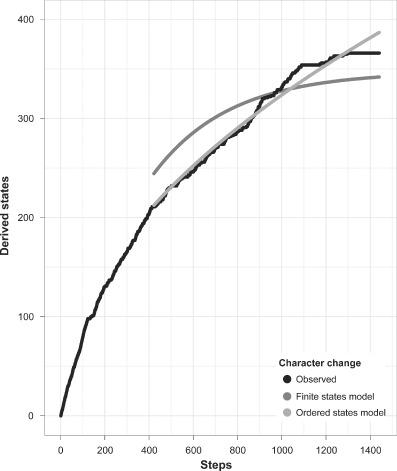
New character states observed along the maximum parsimony morphological phylogeny as a function of the minimal number of steps in the phylogeny (ordered characters were changed to unordered). Two non-linear models fitted to the data are shown in shades of grey.

The segmented regression model fitted to the state:step curve identified step 421 as the breakpoint between slopes, and these initial steps were discarded in subsequent saturation analyses. A Mann-Whitney test rejected the null hypothesis of new character states appearing equally early or late along the curve (W = 87332, *P* = 1.872E−09). Both finite-states and ordered-states non-linear models of character-state accumulation significantly fitted the data (*P* < 2E−16 for all parameters fitted). The finite-states model underestimated the maximum number of states at 347.275 (S.E.M. = 1.132). The ordered-states model estimated the scaling constant at 11.124 (S.E.M. = 0.189877) and the exponent of the power function at 0.488 (S.E.M. = 0.002476). A comparison of the AIC scores of the models revealed essentially no support for the finite-states model (Δ_AIC_ = 2517). The state:step curve, and modeling results are summarized in Fig. 3.

#### (b) Molecular data

About 61% of the mitochondrial data (599 third codon positions and 1734 sites of the loops in the mtrDNA) had very low slopes of <0.2 and were saturated (Table 2), posing a challenge for phylogeny estimation. The *χ*^2^ test for homogeneity of bases implemented in PAUP* was significant for third codon positions of the mitochondrial genes *CYTB* and *COX1*, and the loops of mitochondrial 16S (*P*≤ 0.00086584), and non-significant for every other partition. The overlap between saturated sites and significantly biased base composition requires approaches to reduce the impact of ahistorical signal on estimates of phylogeny. We excluded third positions from *COX1* because the slope of uncorrected/corrected distances indicated complete independence of observed changes from evolutionary changes (Table 2), and down-weighted third positions from *CYTB* and mtrDNA loops in maximum parsimony (MP) and ML analyses of molecular data. This approach was applied in parallel with a traditional all-character equal-weight approach. Since the average saturated site excluding *COX1* third positions had a slope of 0.23 and unsaturated sites an average slope of 0.88 (Table 2), included saturated sites were weighted 25% the value of unsaturated sites. Bayesian analyses did not allow weighting sites, so the down-weighted sites were completely excluded, and we ran parallel analyses of complete and reduced alignments.

**Table 2:** Slope of uncorrected *versus* corrected distances for different loci, codon positions, and rRNA stems or loops

Gene	Positions	Transversions	Transitions
*CYTB*	1^st^	0.74	0.48
	2^nd^	0.89	0.74
	3^rd^	0.24	0.01
*COX1*	1^st^	0.94	0.73
	2^nd^	0.97	0.91
	3^rd^	0.00	0.00
*RAG2*	1^st^	0.95	0.87
	2^nd^	0.96	0.90
	3^rd^	0.82	0.44
12S	Stems	0.91	1.05
	Loops	0.49	0.19
16S	Stems	0.92	1.61
	Loops	0.43	0.02

*CYTB*, mitochondrial cytochrome *b*; *COX1*, mitochondrial cytochrome oxidase I; *RAG2*, recombination activating gene 2.

### (2) Phylogenies

#### (a) Morphological data

##### (i) Parsimony

**Figure 4:**
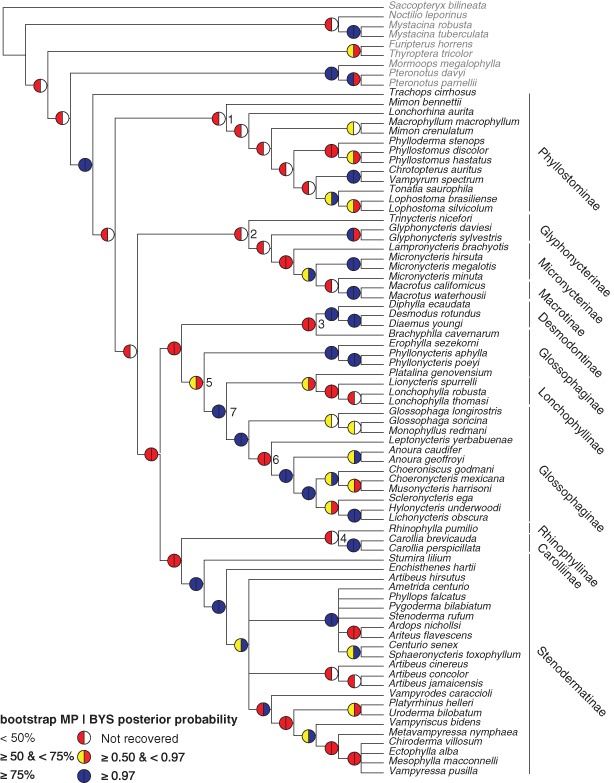
Strict consensus of most parsimonious trees resulting from parsimony analysis of 220 morphological characters (length = 1408; consistency index or CI = 0.2720; retention index or RI = 0.6744), and summary of maximum parsimony (MP) bootstraps and Bayesian (BYS) posterior probabilities. Subfamilies Phyllostominae, Glyphonycterinae, and Micronycterinae correspond to Phyllostominae of Fig. 1. Subfamilies Carolliinae, Rhinophyllinae and Stenodermatinae all feed primarily on fruit. Nodes in conflict with reference phylogeny are numbered, see Table 4. Classification follows Baker *et al.* (2003); outgroups are shown in grey type.

Our analyses recovered 18 most-parsimonious trees of 1408 steps (Fig. 4). Of the 77 clades found in the strict consensus tree, 10 had very strong support with bootstrap values ≥90%. In contrast with previous morphological analyses, Phyllostominae [*sensu*Wetterer *et al.* (2000); Fig. 1] was paraphyletic and split into three lineages (*cf*. Figs 1 and 4). Another notable change was the novel sister relationship of vampire bats (Desmodontinae) with *Brachyphylla cavernarum,* which had low support. This clade appeared as the sister taxon of the nectar-feeding clade comprising Glossophaginae, *Phyllonycteris* + *Erophylla*, and Lonchophyllinae (Fig. 4).

##### (ii) Bayesian

Analyses using the Mkv+Γ model resulted in a poorly resolved 50% majority-rule consensus. Stationarity was achieved after 2500000 generations (one-way ANOVA: F_obs_ = 1.637; *P* = 0.146). Only 65 nodes were recovered in the consensus tree, 82.2% of a fully bifurcating solution. Of these clades, only 25 had Bayesian posterior probabilities ≥0.95, including Desmodontinae, *Phyllonycteris* + *Erophylla*, Glossophaginae + Lonchophyllinae, and Stenodermatinae. However, this might be a very high threshold for the posterior probabilities of branches supported by the smaller sample sizes of morphological data (Yang, 2008; Dávalos & Porzecanski, 2009). Again, the morphological data failed to recover Phyllostominae *sensu*Wetterer *et al.* (2000); Bayesian analyses of our morphological data identified six phyllostomine clades. Fruit feeders, comprising Carolliinae, Rhinophyllinae and Stenodermatinae, were monophyletic. As with MP analyses, *Brachyphylla* was sister to the desmodontine bats (Fig. 4).

#### (b) Molecular data

##### (i) Parsimony

**Figure 5:**
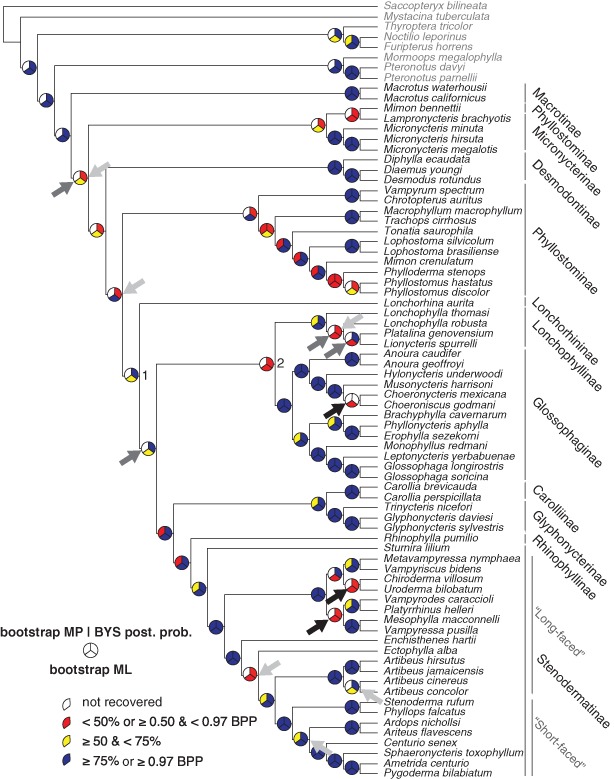
Maximum likelihood (ML) tree resulting from analysis of 6032 molecular characters using a single model of sequence evolution across the entire alignment (1P; log-likelihood = -90656), and summary of ML and maximum parsimony (MP) bootstraps and Bayesian (BYS) posterior probabilities (BPP). Black arrows indicate differences between the 1P ML phylogeny (shown) and the 8b (log-likelihood = -87223; Table 1) multiple-model ML phylogeny (see Section III.2*bii*). Resulting log-likelihoods were higher than those on Table 1 because these searches were not constrained by topology. The resolutions recovered with the 8b partitioned models were *Choeronicus* sister to *Musonycteris*, *Chiroderma* sister to a clade of *Vampyriscus* and *Metavampyressa*, and *Mesophylla* and *Vampyressa* are sister, and sister to a clade of *Uroderma*, *Chiroderma*, *Vampyriscus*, and *Metavampyressa*. Dark grey arrows indicate nodes where ML bootstrap support increased >15% points by partitioning the data into eight models (8b). Light grey arrows indicate shifts across the 0.97 threshold in Bayesian posterior probability between one-partition (shown) and multi-partition models. Arrows pointing up indicate higher BPP with the simpler model (shown) compared to the 8b partition model, and arrows pointing down indicate lower BPP with the model depicted. Nodes in conflict are numbered, see Table 4. Classification follows Baker *et al.* (2003), outgroups are shown in grey type.

**Figure 6:**
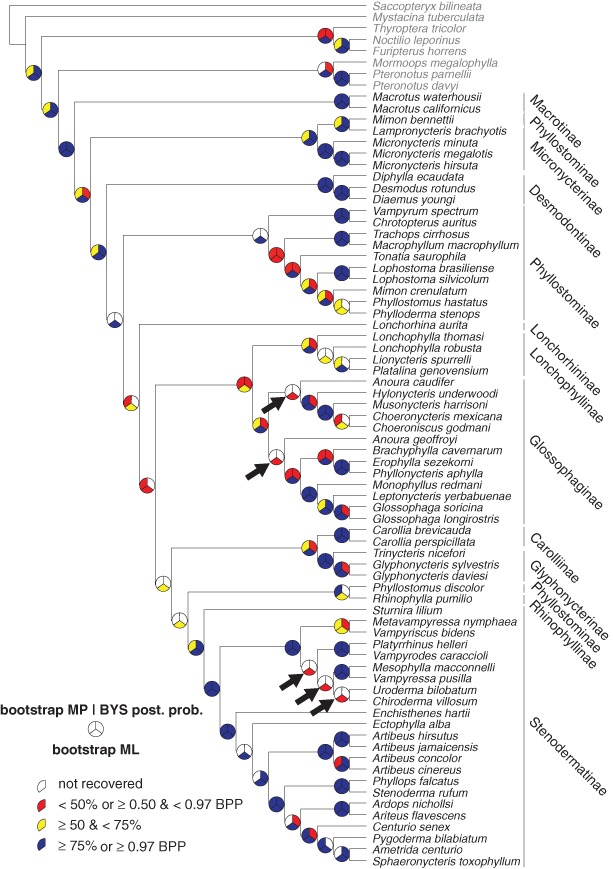
Maximum likelihood (ML) tree resulting from analysis of 5434 molecular characters [excluding mitochondrial cytochrome oxidase 1 (*COX1*) third positions], with weights of 0.25 for loops and mitochondrial cytochrome b (*CYTB*) third positions, and summary of maximum likelihood (ML) and maximum parsimony (MP) bootstraps and Bayesian (BYS) posterior probabilities (BPP). The phylogeny shown was obtained using a single model of sequence evolution across all partitions (model 1P; log-likelihood = −242716). Black arrows indicate differences between the 1P ML phylogeny (shown) and the 8b multiple-model ML phylogeny (Table 1; log-likelihood = −235619; the multiple-model phylogeny makes *Anoura* monophyletic, and provides an alternative resolution for the non-glossophagine nodes highlighted). Classification follows Baker *et al.* (2003), outgroups are shown in grey type.

Our analyses recovered four most-parsimonious trees of 21429 steps (bootstrap support shown in Fig. 5). The tree-wide consistency index or CI is a ratio of the minimum number of steps over observed number of steps that reaches 1 when all characters evolved without homoplasy; while the tree-wide RI sums the per-character RI (see Section II.5). The CI for this tree was 0.2274, and the RI was 0.3804. Excluding completely saturated sites and down-weighting partially saturated sites resulted in 2041 parsimony-informative characters, and four most-parsimonious trees of 8436.50 steps. The strict consensus of those trees was only slightly less well resolved than the strict consensus tree obtained from analyses of all the data (CI = 0.2829; RI = 0.4404; bootstrap support shown in Fig. 6). The latter analyses resulted in a greater number of nodes conflicting with the ML topology, particularly along the backbone of the tree (Fig. 6).

##### (ii) Maximum likelihood

Both the AIC*c* and BIC favoured an eight-partition model (log-likelihood = −87228.14), while the decision-theoretic approach favoured a single partition (log-likelihood = −90681.12). The total number of free parameters and the best-fit model of sequence evolution for each partition selected using the AIC*c* are shown in Table 3. Maximum likelihood analyses running searches with 1 (1P) or 8 (8b) partitions (Table 1) produced fully resolved, strongly supported trees (Fig. 5). Unlike parameter searches when comparing different partitioning schemes, tree searches were not constrained to any topology and converged on higher likelihood solutions than partitioning comparisons (*cf.*Fig. 5 and Table 1). Among the subfamilies recognized by Wetterer *et al.* (2000), we recovered only Desmodontinae, Phyllonycterinae, and Stenodermatinae (*cf*. Figs 1 and 5). The trees resulting from the different partitioning approaches were virtually identical. Both unpartitioned and partitioned trees had roughly similar bootstrap support values in almost all cases, i.e. values differed by ≤5% percentage points. In most instances in which there was a difference >5% points in the bootstrap values, the model with more parameters produced higher support values (10 of 13 instances; Fig. 5).

**Table 3:** Best-fit maximum likelihood (ML) models for each data partition

Partition	Model	Per partition free parameters
Unpartitioned	GTR + I + Γ	11
mtrDNA	GTR + I + Γ	11
mtrDNA stems	GTR + I + Γ	11
mtrDNA loops	GTR + I + Γ	11
12S stems	GTR + I + Γ	11
12S loops	GTR + I + Γ	11
16S stems	GTR + I + Γ	11
16S loops	GTR + I + Γ	11
12S	GTR + I + Γ	11
16S	GTR + I + Γ	11
tRNA^val^	GTR + Γ	10
Pos 1 + 2	SYM + I + Γ	9
Pos 1	GTR + I + Γ	11
Pos 2	HKY + I + Γ	5
Pos 3	GTR+Γ	10
mt pos 1 + 2	HKY + I + Γ	6
mt pos 1	GTR + I + Γ	11
mt pos 2	GTR + I + Γ	11
mt pos 3	HKY + I + Γ	5
*RAG2* pos 1 + 2	GTR + I + Γ	11
*RAG2* pos1	HKY + I + Γ	6
*RAG2* pos 2	HKY + I + Γ	6
*RAG2* pos 3	GTR + Γ	10

The number of free parameters includes six substitution rates under the GTR model (MrBayes can calculate all these, while RAxML estimates five rates relative to the G–T transversion), and three nucleotide frequencies. Γ, discontinuous gamma distribution of rate heterogeneity over sites; GTR, generalized reversible model of sequence evolution; HKY, Hasegawa-Kishino-Yano (Hasegawa *et al.*, 1985) model of sequence evolution, accounting for a transition/transversion rate ratio and unequal base frequencies; mtr, mitochondrial ribosomal partition; pos, positions; mtrDNA, mitochondrial ribosomal DNA; *RAG2*, recombination activating gene 2; SYM, symmetric model of sequence evolution; tRNA^val^, transfer RNA valine.

Similar ML analyses of the molecular data excluding *COX1* third positions and down-weighting *CYTB* third positions and mtrDNA loops resulted in a fully resolved phylogeny with a few key differences (Fig. 6). In particular, support for nodes within Phyllostominae and Glossophaginae decreased markedly, and two genera, *Phyllostomus* and *Anoura*, were rendered polyphyletic (*cf*. Figs 5 and 6).

##### (iii) Bayesian

Analyses using the 1P or 8b partitions and per-partition models of evolution (Table 3) resulted in trees nearly identical to those found in the ML analyses, differing only in relationships within the “long-faced” stenodermatine group (Fig. 5). Stationarity was achieved after 9500000 generations across multiple runs (one-way ANOVA for 1P: *F*_obs_ = 2.369; *P* = 0.050; one-way ANOVA for 8b: *F*_obs_ = 1.430; *P* = 0.221). The majority-rule consensus of post-burn-in trees was fully resolved. Of the 66 ingroup clades recovered, 48 (1P) or 49 (8b) had Bayesian posterior probabilities of ≥0.97, including the subfamilies Desmodontinae, Lonchophyllinae, Phyllonycterinae, and Stenodermatinae. Support values were largely similar between the two partitioning approaches. Posterior probability values changed across the 0.97 BPP-threshold depending on the partitioning scheme in only five instances, and mostly in the direction of more support when more parameters were fitted to the data (Fig. 5).

Similar analyses of a reduced molecular data matrix (3,703 nucleotides, loops and mt third positions excluded) resulted in a more poorly resolved and supported tree (Fig. 6). In particular, nodes along the backbone of the tree, among them Phyllostominae, Glossophaginae and Lonchophyllinae, and Stenodermatinae, had lower support or conflicting resolution relative to analyses of all the molecular data (*cf*. Figs 5 and 6). As in the ML analyses of the reduced matrix, *Phyllostomus* was not monophyletic (Fig. 6), but *Anoura* was monophyletic with low support (0.90 BPP in 1P analyses, and 0.84 BPP in 8b analyses, not shown).

#### (c) Combined data

##### (i) Parsimony

**Figure 7:**
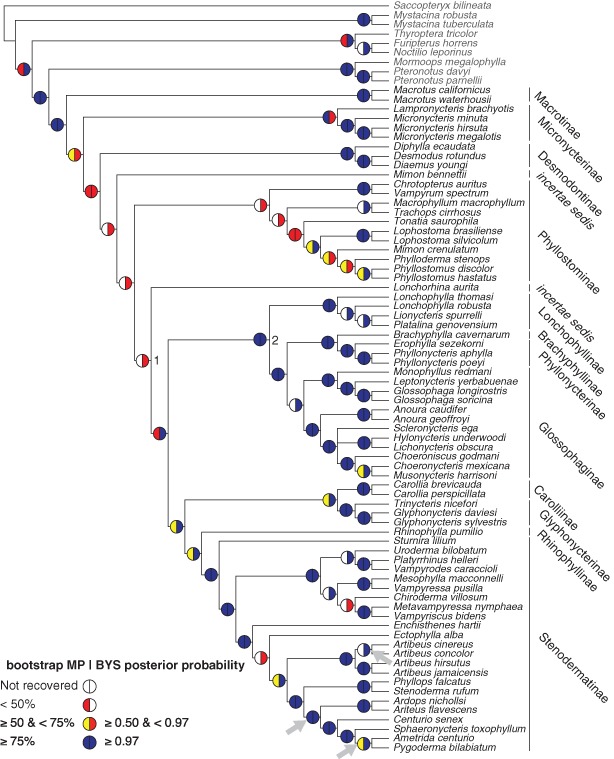
Fifty percent majority-rule consensus of Bayesian trees resulting from analysis of 6032 molecular and 220 morphological characters using one model of evolution for variable morphological characters, and separate models for eight partitions of the molecular data (8b + morphology runs, harmonic mean of log-likelihood = -92829), and summary of maximum parsimony (MP) bootstraps and Bayesian (BYS) posterior probabilities (BPP). Light grey arrows pointing up indicate <0.97 BPP with the simpler model compared to the morphology + 8b partition model (shown). Nodes in conflict with the reference phylogeny are numbered, see Table 4. Classification follows Baker *et al.* (2003), outgroups are shown in grey type.

**Figure 8:**
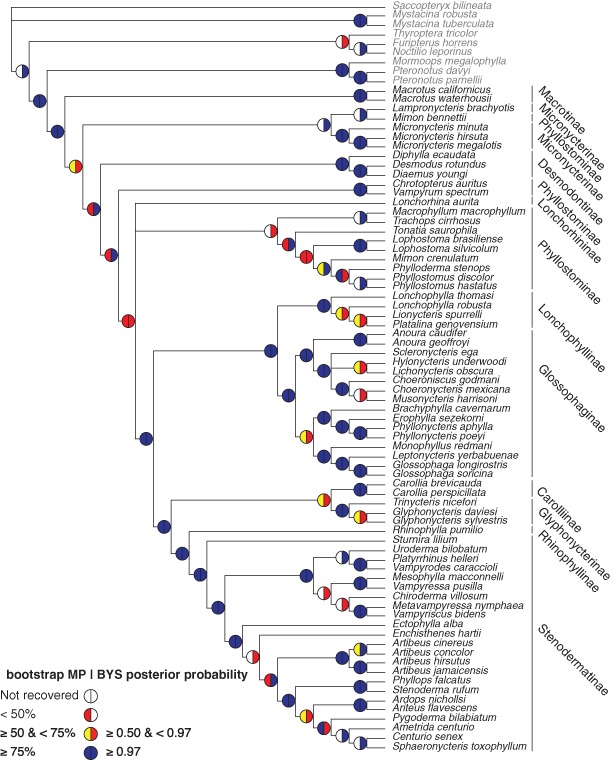
Fifty percent majority-rule consensus of Bayesian trees resulting from analysis of 3699 molecular (excluding mitochondrial loops and third positions) and 194 morphological characters (excluding characters thought to be convergent) using one model of evolution for variable morphological characters, and separate models for seven partitions of the molecular data (8b + morphology runs, harmonic mean of log-likelihood =−33307), and summary of maximum parsimony (MP) bootstraps and Bayesian (BYS) posterior probabilities (BPP). Classification follows Baker *et al.* (2003), outgroups are shown in grey type.

Our analyses recovered two most-parsimonious trees of 23006 steps (CI = 0.2284; RI = 0.4057; Fig. 7). The MP analysis supported the monophyly of only 6 of the 18 higher-level clades recognized by Wetterer *et al.* (2000). The backbone of the phylogeny was especially poorly supported and these relationships generally garnered <50% bootstrap support. Analyses down-weighting loops and *CYTB* third codon positions, excluding *COX1* third positions and morphological characters as explained in Section III.4*a*, resulted in a less-resolved tree (Fig. 8).

##### (ii) Bayesian

Results of the Bayesian analyses for the concatenated data set under both partitioning schemes and the models presented in Table 3 were identical to one another, save for the placement of *Mimon bennettii*. Stationarity was achieved after 14500000 generations for both the two-model (one morphological and one molecular: one-way ANOVA *F*_obs_ = 1.856, *P* = 0.073) and nine-model runs (one morphological and eight molecular; one-way ANOVA *F*_obs_ = 2.277, *P* = 0.059). The majority-rule consensus of post-burn-in trees was well resolved and strongly supported across multiple hierarchical levels, with only one unresolved ingroup node (Fig. 7). Overall, more than 80% of the relationships found in the MP tree also appeared in the BYS tree, with exceptions along the backbone, among “phyllostomines,” and within specific lower clades (Fig. 7). The former “Phyllostominae”: Macrotinae, Micronycterinae, Phyllostominae, Lonchorhininae, and Glyphonycterinae were each monophyletic and did not form a clade; predominantly nectar-feeding taxa formed a clade (i.e. Glossophaginae, Brachyphyllinae, Phyllonycterinae, and Lonchophyllinae); and the genera *Carollia* and *Rhinophylla*, both once included in Carolliinae, did not form a clade. *Mimon bennettii* was not closely related to any of the former “phyllostomine” clades.

Analyses of the reduced molecular matrix (see above) and a reduced morphological matrix resulted in a less resolved tree (*cf*. Figs 7 and 8). Despite this, some nodes were better supported with the reduced data, notably along the backbone of the phylogeny.

### (3) Incongruence analyses

#### (a) Support for nodes conflicting with reference phylogeny

A survey revealed low support from the morphological and molecular data for the majority of conflicting nodes (Table 4). The down-weighting/exclusion of saturated sites and exclusion of potentially convergent morphological characters (below) generally decreased support for those nodes.

**Table 4:** Support for nodes conflicting with the reference phylogeny (Baker *et al.*, 2003)

Node defined by MRCA of taxa below	Data	Method	Figure	Node	Support	Unambiguous character transformations (diet)	Revised support
*Mimon bennettii* and *Lophostoma silvicolum*	Morphology	MP	4	1	6.0%	4 (0)	10%^1^
*Trinycteris nicefori* and *Macrotus waterhousii*	Morphology	MP	4	2	11.2%	4 (2)	2%^2^
*Brachyphylla cavernarum* and *Diaemus youngi*	Morphology	MP, Bayesian	4	3	24.1%, 0.60	3 (1)	13%, 0.84
*Rhinophylla pumilio* and *Carollia perspicillata*	Morphology	MP	4	4	40.2%	4 (2)	45%
*Erophylla sezekorni* and *Lichonycteris obscura*	Morphology	MP, Bayesian	4	5	**92%**, 0.59	9 (7)	–,–^3^
*Leptonycteris yerbabuenae* and *Lichonycteris obscura*	Morphology	MP, Bayesian	4	6	49.4%, 0.85	2 (2)	–, 0.76
*Lonchorhina aurita* and *Pygoderma bilabiatum*	Molecular	ML, Bayesian	5	1	**73%**, **0.98**	–	**62%**,–
*Lonchophylla thomasi* and *Erophylla sezekorni*	Molecular	ML, Bayesian	5	2	48%, **0.83**	–	**51**%, 0.53
*Lonchorhina aurita* and *Pygoderma bilabiatum*	Combined	Bayesian	7	1	0.85	–	0.70
*Lonchophylla thomasi* and *Musonycteris harrisoni*	Combined	MP, Bayesian	7	2	**89%**, **1.00**	–	**76%**, **1.00**

Revised support corresponds to analyses excluding morphological characters hypothesized to be linked to diet specialization, and excluding and/or down-weighting saturated molecular data. A dash (–) indicates the node was not recovered in that analysis. Support from molecular and combined data was obtained with the single-partition analyses of the molecular data. ML, maximum likelihood; MP, maximum parsimony; MRCA, most recent common ancestor. Values in bold highlight ≥50% bootstrap support or ≥0.97 Bayesian posterior probability.

1 Clade defined by this node includes all genera of ‘Phyllostominae’*sensu*Wetterer *et al.* (2000).

2 Clade defined by this node is a subset of the clade of footnote 1.

3 MRCA *Platalina genovensium* and *Lichonycteris obscura* (Fig. 4, node 7) = ≤50%/0.89, *Brachyphylla*, *Erophylla*, and *Phyllonycteris* formed a clade with the desmodontines.

#### (b) Distribution of retention index of different data partitions

**Figure 9:**
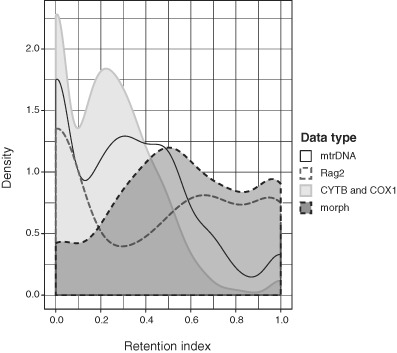
Density of the frequency distribution of per-character retention index (RI) from individual data partitions optimized on the reference phylogeny of Fig. 2. *CYTB*, mitochondrial cytochrome *b*; *COX1*, mitochondrial cytochrome oxidase I; mtrDNA, mitochondrial ribosomal DNA; *RAG2*, recombination activating gene 2.

**Figure 10:**
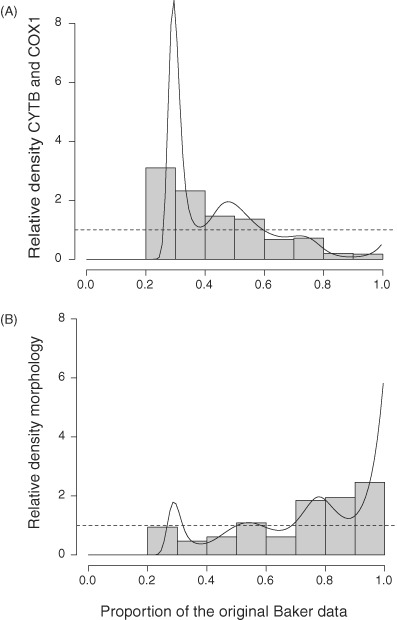
Densities of the distributions of per-character retention index (RI) from: (A) mitochondrial coding and (B) morphological data relative to the original Baker *et al.* (2003) data [i.e. mitochondrial ribosomal (mtr) and *RAG2* DNA sequences]. Solid bars show the observed relative density, smoothed in the black line. The dashed line indicates the expected relative density if the frequency distributions of RIs from the *y*-axis data and the Baker *et al.* (2003) data were identical. *CYTB*, mitochondrial cytochrome b; *COX1*, mitochondrial cytochrome oxidase 1.

The density of the distributions of the retention index from different data types showed that both the mtrDNA and *RAG2* data sets used to generate the reference phylogeny had peaks of low values (Fig. 9). Substantial portions of the mtrDNA and *RAG2* data were homoplastic and in conflict with the resulting phylogeny. This peak at low values was also observed with the *CYTB* and *COX1* data, which were not used to generate the reference phylogeny. By contrast, the peak in the frequency distribution of the morphological data was at intermediate RI values. Kolmogorov-Smirnov tests comparing the distributions of RIs from the mitochondrial coding data (D = 0.1558, *P* = 3.266E−13) and the morphological characters (D = 0.412, *P* < 2.2E−16) to the original Baker *et al.* (2003) data showed significant differences. The location of those differences is shown in Fig. 10. Compared to the original data, the RIs of the mitochondrial coding data have a much higher frequency of values of around 0.3, while the morphological data have a disproportionate amount of character state changes interpreted as perfectly homologous throughout the tree. Among 150 characters with RI = 1, there were three times as many morphological characters, and twice as many *RAG2* characters as expected if all character types were equally distributed (G-test of goodness-of-fit G_3_ = 96.172, *P* = 1.03E−20). Character classes within morphology were roughly equally represented among the 37 characters with RI = 1 (G-test of goodness-of-fit G_5_ = 7.458, *P* = 0.189).

#### (c) Partitioned likelihood support

**Figure 11:**
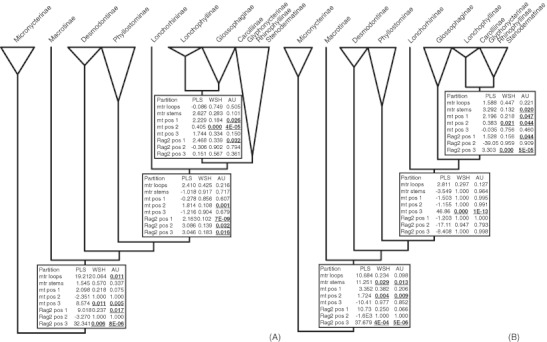
Partitioned likelihood support (PLS) resulting from maximum likelihood analyses of concatenated mitochondrial ribosomal (mtr) and coding sequences, and nuclear data. Significant support of a given partition for the node (in bold) was estimated using the weighted Shimodaira-Hasegawa (WSH) and the approximately unbiased (AU) tests. The nodes examined were incongruent with analyses of mtr and nuclear data by Baker *et al.* (2003). (A) Results of maximum likelihood (ML) analysis using one partition (1P, log-likelihood = −81953), all model parameters were equal across partitions when calculating PLS. (B) Results of ML analysis using eight partitions (8b, log-likelihood = −78705), all parameters but the phylogeny were allowed to vary across partitions when calculating PLS. Mt, mitochondrial; mtrDNA, mitochondrial ribosomal DNA; *RAG2*, recombination activating gene 2.

Maximum likelihood analyses of these data under the 1P and 8b partition schemes yielded two alternative resolutions, both of which differed from all-taxa analyses in placing Micronycterinae as sister to all other phyllostomids (*cf*. Figs 5 and 11). We focused PLS analyses on the placements of Micronycterinae and *Lonchorhina*, and the relationships among nectar-feeding bats in the subfamilies Glossophaginae and Lonchophyllinae because each of these conflicts with the reference phylogeny (Fig. 2). Support for each of these nodes from individual partitions is summarized in Fig. 11.

#### (d) ML-based tree comparisons

A total of 45 taxa overlapped across all data sets and prior phylogenies (see TreeBASE accession). Table 5 summarizes results of the WSH and AU tests of differences in log-likelihood of the data given the set of compared phylogenies. In general, the WSH test identified fewer significant conflicts than the AU test. Instances of disagreement between the tests clustered around the fit of the protein-coding genes to different trees and in every case involved a significant AU test and a non-significant (*P* > 0.100) or marginally significant (0.05 *> P* ≤ 0.100) WSH test (Table 5). The molecular data and their partitions significantly rejected the morphology-derived hypotheses of Wetterer *et al.* (2000) (*P* ≤ 2E−07), and all molecular data rejected the current morphological resolution (*P* ≤ 4E−06). Individual molecular data sets—mtrDNA, protein-coding mitochondrial DNA, and *RAG2*—marginally rejected the phylogeny of Baker *et al.* (2003) (*P* ≥ 0.080). Individual molecular data sets of different genomic origin were incompatible with one another: *RAG2* rejected the mtrDNA phylogeny (*P* ≤ 0.044), the mtrDNA data rejected the *RAG2* phylogeny (*P* ≤ 1E−09), and the mitochondrial protein-coding data marginally rejected the *RAG2* phylogeny (*P* ≤ 0.057).

**Table 5:** Significance of difference in log-likelihoods of alternative phylogenies using different data partition schemes and data sets, estimated using the weighted Shimodaira-Hasegawa (Shimodaira & Hasegawa, 1999) and approximately unbiased (Shimodaira, 2002) tests

Phylogeny	Molecular 8b partitioned		Baker partitioned		mtrDNA partitioned		*COX1* & *CYTB*		*RAG2*	
	WSH	AU	WSH	AU	WSH	AU	WSH	AU	WSH	AU
Wetterer *et al.* (2000)	0	2E–07	0	0	0	0	0	0	0	0
MP morphology	0	6E–06	0	4E–04	0	0	0	0	0	0
MP molecular	0.001	4E–06	0.001	1E–04	0.006	3E–04	0.144	0.016	0.012	2E–04
MP combined	0.007	5E–04	0.013	0.001	0.059	0.006	0.916	0.523	0.043	0.005
ML molecular 1P	0.998	0.735	0.998	0.803	0.993	0.675	0.893	0.422	0.139	0.051
ML molecular 8b	0.884	0.394	0.760	0.278	0.843	0.292	0.881	0.382	0.147	0.008
ML mitochondrial	0.986	0.643	0.972	0.642	0.989	0.602	0.624	0.133	0.160	0.060
ML mtrDNA partitioned	0.226	0.074	0.751	0.295	0.999	0.990	0.279	0.074	0.044	0.004
ML *RAG2*	0.032	0.008	0.125	0.036	0	0	0.057	0.011	0.999	0.990
Baker *et al.* (2003)	0.484	0.157	0.676	0.290	0.307	0.080	0.566	0.221	0.075	0.008
Bayesian morphology	0	6E–09	0	0	0	0	0	0	0	0
Bayesian combined 8b + morphology	0.935	0.469	0.879	0.422	0.913	0.417	0.998	0.824	0.121	0.009

The likelihoods of protein-coding data were analyzed using a codon model. 1P, one partition for molecular data; AU, approximately unbiased text; *CYTB*, mitochondrial cytochrome *b*; *COX1*, mitochondrial cytochrome oxidase I; ML, maximum likelihood; MP, maximum parsimony; mtrDNA, mitochondrial ribosomal DNA; *RAG2*, recombination activating gene 2; WSH, weighted Shimodaira-Hasegawa test.

Results of comparisons of phylogenies obtained excluding potentially convergent morphological characters and *COX1* third codon positions, and down-weighting (MP and ML) or excluding (Bayesian) *CYTB* third codon positions and mtrDNA loops are shown in Table 6. These comparisons included 50 taxa that overlapped across all compared phylogenies [i.e. there were more taxa because the Baker *et al.* (2003) and Wetterer *et al.* (2000) trees were not compared, see TreeBASE accession]. Although the molecular data rejected the results of MP analyses of all kinds of data, there were fewer significant conflicts between molecular data partitions and phylogenies derived from the combined data. For example, the *RAG2* partition did not reject the combined Bayesian phylogeny derived from the reduced character set (Fig. 8, *P*≥ 0.528, Table 6), but rejected the phylogeny derived from all characters (Fig. 7, *P*≥ 0.009, Table 5).

**Table 6:** Significance of difference in log-likelihoods of phylogenies generated by excluding or down-weighting saturated molecular characters and putatively convergent morphological characters

Phylogeny	Molecular 8b partitioned^1^		Baker partitioned^2^		mtrDNA partitioned		*COX1* and *CYTB*		*RAG2*	
	WSH	AU	WSH	AU	WSH	AU	WSH	AU	WSH	AU
MP morphology^3^	0	0	0	1E–06	0	4E–07	0	4E–05	0	4E–05
MP molecular^4^	9E–05	0	2E–04	2E–04	0	0	0.005	2E–05	0.002	3E–05
MP combined^5^	0	2E–09	9E–05	1E–07	0	0	0	0	0.001	0.001
ML molecular 8b^4^	0.683	0.260	0.798	0.330	0.999	0.807	0.780	0.254	0.929	0.547
ML mtrDNA partitioned^6^	0.358	0.068	0.389	0.066	0.900	0.980	0.902	0.438	0.009	4E–04
Bayesian DNA 8b^1^	0.794	0.383	0.898	0.519	0.064	0.002	0.041	0.001	0.934	0.417
Bayesian combined 8b + morphology^7^	0.994	0.796	0.981	0.697	0.661	0.214	0.937	0.496	0.932	0.528

Results of the weighted Shimodaira-Hasegawa (Shimodaira & Hasegawa, 1999) test are shown on the left of each pair, and of the approximately unbiased test (Shimodaira, 2002) test on the right of each pair. The likelihoods of protein-coding data were analyzed using a codon model. AU, approximately unbiased text; *CYTB*, mitochondrial cytochrome *b*; *COX1*, mitochondrial cytochrome oxidase I; ML, maximum likelihood; MP, maximum parsimony; mtrDNA, mitochondrial ribosomal DNA; *RAG2*, recombination activating gene 2; WSH, weighted Shimodaira-Hasegawa test.

1 Excluded mtrDNA loops and mt third codon positions.

2 Excluded mtrDNA loops.

3 Excluded putatively convergent morphological characters.

4 Excluded *COX1* third codon positions; down-weighted *CYTB* third codon positions and mtrDNA loops.

5 Excluded *COX1* third codon positions and putatively convergent morphological characters; down-weighted *CYTB* third codon positions and mtrDNA loops.

6 Down-weighted mtrDNA loops.

7 Excluded mtrDNA loops, mt third codon positions, and putatively convergent morphological characters.

### (4) Analyses of adaptive convergence

#### (a) Morphological data

Based on both the reference phylogeny and new molecular analyses, some morphology-supported clades were incorrect (Table 4, *cf*. Figs 2, 4 and 5). Support for these clades was low in our morphology trees (Table 4; <75% MP bootstrap, and <0.97 BPP), and tended to spuriously unite groups with similar feeding strategies. For example, gleaning insectivory is shared across *Lophostoma*, *Macrotus, Micronycteris*, *Tonatia*, *Trinycteris*, *Lampronycteris*, and *Glyphonycteris* (Bell & Fenton, 1986; Kalka & Kalko, 2006), nectar feeding is shared across Glossophaginae and Lonchophyllinae (Coelho & Marinho-Filho, 2002; Zortea, 2003), and *Carollia* and *Rhinophylla* both feed on *Piper* (Thies, Kalko & Schnitzler, 1998; Henry & Kalko, 2007) (Table 4, Fig. 4). The morphological support for these putative clades, together with the ecological relevance of these groups, suggested that adaptive convergence might underlie recovery of these relationships in morphological trees.

We examined unambiguous character transformations on the morphological tree along the branches defining the conflicting nodes to investigate whether derived traits associated with feeding ecology were driving the potentially spurious relationships. The nodes involving the nectar-feeding subfamilies Glossophaginae and Lonchophyllinae were the best supported (Table 4 and Fig. 4), and had a majority of character transformations occurring in characters associated with the feeding apparatus. Examples of unambiguous changes inferred at those nodes include the loss of lobes at incisor margins, the acquisition of a brush of hair on the tongue, increases in the number of papillae on the tongue, and changes in the arrangement of hyoid musculature. All of these features are thought to be specializations for nectarivory (Griffiths, 1982; Freeman, 1995; Carstens, Lundrigan & Myers, 2002).

To investigate the potential convergence of morphological data supporting relationships between and within Glossophaginae and Lonchophyllinae (*sensu*Baker *et al.*, 2003; Fig. 2), we mapped the suite of 32 feeding characters that unambiguously supported either or both Glossophaginae + Lonchophyllinae and Glossophaginae + Lonchophyllinae + Phyllonycterinae *sensu*Wetterer *et al.* (2000) onto the reference phylogeny. In addition, we identified several characters that might be related to gleaning insectivory or *Piper* frugivory and examined their distribution on the reference phylogeny as well. These two sets of characters were not mutually exclusive, and added up to 36 characters. Because Glossophaginae and Lonchophyllinae *sensu*Baker *et al.* (2003) are paraphyletic branches in a larger monophyletic “plant-visiting” clade (Fig. 2), it could be that some character transformations occurred in an ancestral lineage and are therefore plesiomorphic in the nectar-feeding clades. By contrast, changes to similar states along each of the branches leading to glossophagines and lonchophyllines would indicate adaptive convergent evolution.

Of the 29 cases where states were shared between the glossophagine and lonchophylline clades (*sensu*Baker *et al.*, 2003), we found equally parsimonious alternative optimizations on the reference phylogeny for 15 characters, including features of the pelage and integument, tongue, hyoid musculature, dentition and cranium. These character states were interpreted as either primitive retentions or convergence depending on whether accelerated (ACCTRAN) or delayed transformations (DELTRAN) were used. Nine states were primitively retained and five states were convergent no matter which optimization was used.

Since the morphological character analyses revealed a majority of ambiguous optimizations, we researched the evolution of nectarivory itself, as feeding niche and skull morphology have coevolved in phyllostomids (Freeman, 1995, 2000; Dumont *et al.*, 2012). Two scenarios require the same number of steps in parsimony optimization: (*i*) nectarivory evolved once and then was lost, or (*ii*) it evolved twice from non-nectarivore ancestors. Although lacking an explicit character analysis, the most recent analyses of nectar-feeding lineages interpreted the phylogeny to imply that nectarivory evolved twice (Datzmann *et al.*, 2010). Bayesian character mapping of the evolution of feeding ecology that accounted for branch lengths has corroborated this interpretation, with overwhelming support for a model in which nectarivory evolved twice (Rojas *et al.*, 2011).

The independent evolution of specialized nectar-feeding structures is reflected by observations of morphological character construction. In the subset of characters that unambiguously supported the clade that included Glossophaginae, Lonchophyllinae and Phyllonycterinae *sensu*Wetterer *et al.* (2000), character definition affected interpretation of homology. For example, we chose to define a single character for the presence of a brush tip on the tongue, emphasizing the similarities among these three subfamilies, with subsequent characters describing differences in shape and distribution of papillae. As noted in the methods section, we coded taxa lacking a brush tip as ‘-.’ By contrast, Griffiths (1982, 1983) defined those characters to highlight the differences among the tongues, creating two separate presence/absence characters; one character for the presence of a groove lined with hair-like papillae, a morphology seen among the lonchophylline species, and the second for the presence of a hair-like brush-tip, a morphology seen among the glossophagine and phyllonycterine species. There have been objections raised to this type of character definition (Smith & Hood, 1984), and we chose to emphasize the primary homology of the hair-like papillae among the taxa that have them. This choice, which we preferred on philosophical grounds, along with many others made during the character definition process, affected the outcome of our analysis. We mention the alternative approach of Griffiths (1982, 1983) to note that there are anatomical reasons to question the homology of some of the characters that support a clade of all nectar-feeding bats, independent of subsequent molecular studies.

The most striking result of morphological character optimization on the reference phylogeny was the considerable homoplasy required by all possible optimizations of many of these characters given the structure of Glossophaginae *sensu*Baker *et al.* (2003). Unlike traditional phylogenies based on morphology that have placed *Brachyphylla* and *Erophylla* in their own subfamilies outside of a more restricted Glossophaginae *sensu*Wetterer *et al.* (2000), both the reference phylogeny and our combined analyses (Fig. 7) suggest that these taxa nest within the glossophagine clade, together with *Phyllonycteris*, as the sister taxa to *Glossophaga*, *Monophyllus*, and *Leptonycteris*. Given this resolution, states that are shared between the *Choeronycteris*-allied clade and the *Glossophaga*-allied clade often require convergent evolution or reversal in the *Brachyphylla* + *Erophylla* clade. This occurred in 13 of the 29 characters whose optimizations we examined.

The mostly ambiguous optimizations of this subset of characters, the lability of the states within Glossophaginae *sensu*Baker *et al.* (2003), and the possible re-interpretation of character states, all support the hypothesis that nectarivory and its specialized structures have evolved twice (Datzmann *et al.*, 2010; Rojas *et al.*, 2011). To investigate the effect of including functionally convergent characters, we reanalyzed the morphological data excluding characters that both: (*i*) unambiguously supported spurious clades, and (*ii*) were related to feeding ecology (i.e. concerning dentition, tongue structure and musculature); results are shown in Table 4. The exclusion of these characters reduced morphological support for the potentially spurious clades with the exception of the clade comprising *Brachyphylla* and the blood-feeding Desmodontinae (Table 4).

#### (b) Molecular data

Two clades supported by the molecular data were identified as potentially incorrect based on the reference phylogeny: a nectar-feeding clade consisting of Glossophaginae and Lonchophyllinae, and a clade defined by the position of *Lonchorhina* (Table 4, *cf*. Figs 2 and 5). Support for the nectar-feeding clade was low, while support for the position of *Lonchorhina* was higher (Table 4). The nectar-feeding clade was supported primarily by substitutions in the first and second codon positions in the mitochondrial protein-coding genes (Fig. 11A). The provenance of support for the position of *Lonchorhina* seemed to depend on the resolution among nectar-feeding subfamilies. Mitochondrial third codon positions provided significant support when nectar-feeding subfamilies did not form a clade (Fig. 11B), and the exclusion and down-weighting of these sites tended to reduce support, or break up the node altogether (Table 4). Adaptive convergence cannot explain the position of *Lonchorhina* in our analyses because: (*i*) support for the clade arises mostly from synonymous substitutions at third codon positions and declines/disappears when saturation at these sites is accounted for (Table 4); and (*ii*) there is no clear link between the potentially incorrect clade and a shared ecological function selecting for a given genotype (e.g. dietary specialization). For these reasons, we propose that saturation better accounts for the position of *Lonchorhina* than evolutionary convergence.

By contrast, a spurious nectar-feeding clade fits the criteria for hypothesizing adaptive convergence: (*i*) although weak, support rose slightly when accounting for saturated sites (Table 4, *cf*. Figs 5 and 6); (*ii*) analyses of a greater sample of nuclear data has confirmed that this clade is spurious (Datzmann *et al.*, 2010); (*iii*) support for the spurious clade is localized in nonsynonymous substitutions in mitochondrial protein-coding genes (Fig. 11); and (*iv*) there is a clear link between the clade and a shared ecological function, nectar-feeding. We investigated a shift in selection pressure as the mechanism underlying the convergent evolution of the mitochondrial protein-coding genes. We conducted these tests on the 56-species subset of taxa for which all partitions were available, and used the reference phylogeny as underlying evolutionary relationships, i.e. the nectar-feeding subfamilies Glossophaginae and Lonchophyllinae did not form a clade.

We found a shift toward a lower *K*_*a*_*/K*_*s*_ in each of the nectar-feeding clades relative to all other phyllostomid bats (Table 7). Differential selection was inferred for 25 codons of the *CYTB* gene in each nectar-feeding clade, and 24 of those were common to both clades. To investigate whether this shift toward more strongly negative selection might be associated with an earlier ecological change, we fitted the shifting *K*_*a*_*/K*_*s*_ model to a clade of “plant-visiting” phyllostomids (descendants of the most recent common ancestor of *Erophylla* and *Artibeus* in Fig. 2). A shift to a lower *K*_*a*_*/K*_*s*_ was estimated for 26 codons of the *CYTB* gene in the plant-visiting clade (Table 7), and all codons undergoing differential selection matched codons identified as undergoing differential selection in analyses of nectar-feeding clades relative to all phyllostomids. Based on these results, we re-estimated the *K*_*a*_*/K*_*s*_ ratios for different site classes among the nectar-feeding clades relative to the plant-visiting clade, rather than to the entire family. We uncovered significant shifts toward higher *K*_*a*_*/K*_*s*_ ratios for both clades at 18 codons, with three additional codons identified for Glossophaginae. In every case the shifts were inferred for codons that were earlier identified as shifting to lower *K*_*a*_*/K*_*s*_ ratios in plant-visiting phyllostomids. Compared to other plant-visiting bats, the *K*_*a*_*/K*_*s*_ ratios of nectar-feeding clades were significantly higher even when analyzing both clades simultaneously (Table 7). Because tests comparing plant-visiting bats to other phyllostomids revealed shifts to lower *K*_*a*_*/K*_*s*_ ratios, the shift to higher *K*_*a*_*/K*_*s*_ ratios in nectar-feeders cannot be considered plesiomorphic among these lineages.

**Table 7:** Tests of shift in selection pressure in glossophagine and lonchophylline clades *sensu*Baker *et al.* (2003) relative to other phyllostomids in mitochondrial protein-coding sequences mapped onto the reference phylogeny

Model	Log-likelihood	*K*_*a*_*/K*_*s*_	Branch in phylogeny	*P* value
Clade model C	−27443.88	0.10568	All	1E–251
		0.00346	Other phyllostomids	
		0.00120	Glossophaginae	
Clade model C	−27442.26	0.10475	All	2E–252
		0.00331	Other phyllostomids	
		0.00000	Lonchophyllinae	
Clade model C	−27436.44	0.10894	All	7E–255
		0.00492	Other phyllostomids	
		0.00184	Plant-visiting phyllostomids	
Nearly neutral	−28025.00	0.01483	All (97.3% of sites)	Null model
		1.00	All (2.7% of sites)	
Clade model C	−16315.16	0.00287	All plant-visiting phyllostomids	2E–117
		0.12379	Other plant-visiting phyllostomids	
		0.16786	Glossophaginae	
Clade model C	−16314.49	0.00299	All plant-visiting phyllostomids	1E–117
		0.12876	Other plant-visiting phyllostomids	
		0.21523	Lonchophyllinae	
Clade model C	−16312.42	0.00297	All plant-visiting phyllostomids	1E–118
		0.11759	Other plant-visiting phyllostomids	
		0.18254	Lonchophyllinae and Glossophaginae	
Nearly neutral	−16586.75	0.01121	All plant-visiting (97.3% of sites)	Null model
		1.00	All plant-visiting (2.7% of sites)	

The model of varying ratio of non-synonymous to synonymous (*K*_*a*_*/K*_*s*_) along branches (clade model C) fits three site classes to the data: *K*_*a*_*/K*_*s*_ < 1 (‘All’), *K*_*a*_*/K*_*s*_ = 1, and *K*_*a*_*/K*_*s*_ varying between background (‘Other’) and foreground (named) branches (Bielawski & Yang, 2004). No codons were found to belong to the neutral site class. The null nearly-neutral model fits two site classes to all branches: *K*_*a*_*/K*_*s*_ < 1, and *K*_*a*_*/K*_*s*_ = 1.

**Figure 12:**
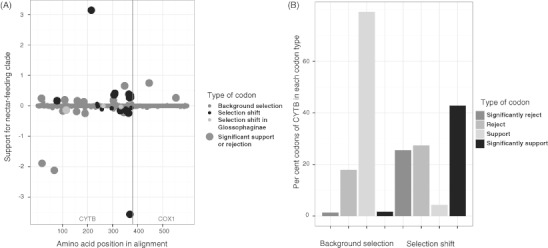
Relationship between estimates of the ratio of non-synonymous to synonymous substitutions (*K*_*a*_*/K*_*s*_) in the Glossophaginae and Lonchophyllinae subfamilies, and support for the spurious nectar-feeding clade in the mitochondrial protein-coding alignment. (A) Support for nectar-feeding clade from amino acid substitutions along the entire mitochondrial protein-coding alignment [mitochondrial cytochrome b (*CYTB*) and mitochondrial cytochrome oxidase 1 (*COX1*). The mean log-likelihood at codons inferred to have shifted to higher *K*_*a*_*/K*_*s*_ in the nectar feeders did not differ for the alignment as whole (*P* = 0.339), or for *CYTB* (*P* = 0.694). (B) Relative frequency of codons among support or rejection extremes. Extreme support or rejection was significantly associated with the inferred selection shift (*P*-value = 7.402E–12).

**Figure 13:**
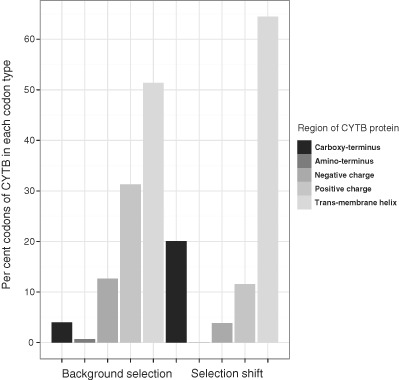
Relative frequency of codons under different inferred ratio of non-synonymous to synonymous substitutions (*K*_*a*_*/K*_*s*_) in Glossophaginae and Lonchophyllinae across mitochondrial cytochrome b (*CYTB*) functional regions. Specific functional protein regions were significantly associated with the inferred selection shift (*P* value = 0.01778).

To investigate the connection between support for the incorrect clade and the shift in *K*_*a*_*/K*_*s*_ in nectar-feeding lineages relative to other plant-visiting phyllostomids (Table 7), we compared the site likelihoods of the translated mitochondrial sequences under trees with and without the potentially spurious clade (as in Fig. 11). If the support for the incorrect clade was connected to shifts in *K*_*a*_*/K*_*s*_ in the two independent lineages, then the sites under differential selection should provide the strongest signal for the nectar-feeding clade. We fitted these models using the mammalian mitochondrial model of protein evolution (MTMAM; Yang, Nielsen & Hasegawa, 1998) implemented in RAxML, and generated a null distribution of the difference in per-site log-likelihoods by simulating codons using the *evolver* function in PAML, translating them into protein sequences, and fitting them to the phylogenies using RAxML (Fig. 12A). Sites under shifting selection were significantly associated with outliers both in extreme support and extreme rejection of the spurious nectar-feeding clade (Fig. 12B) relative to the null distribution of differences between alternative phylogenies (G-tests of goodness-of-fit for both *CYTB* and *COX1* G_2_ = 59.182, *P* = 1.409E–13; for *CYTB* only G_2_ = 51.258, *P* = 7.402E–12). That is, codons under differential selection were overrepresented among both synapomorphies (support) and autapomorphies (rejection) of the nectar-feeding clade. The association between shifting selection sites and both high- and low-outliers of support resulted in non-significant comparisons of likelihood for the two site classes (one-way ANOVA for the *CYTB* and *COX1* alignment *F*_obs_ = 0.917, *P* = 0.339; for *CYTB* alone *F*_obs_ = 0.405, *P* = 0.694). Finally, we mapped the location of the amino acids under shifting selection pressures based on the structural model of Esposti *et al.* (1993). Sites inferred to be under shifting selection were also significantly associated with specific regions of the protein (G-test of independence G_4_ = 11.942, *P* = 0.018), particularly the carboxy-terminus and trans-membrane regions (Fig. 13).

## IV. DISCUSSION

We can classify drivers of phylogenetic incongruence into two broad categories: methodological and biological. In the first category are analytical choices, such as taxonomic sampling, the number of characters available, and the optimization algorithm applied. In the second category are biological processes that may result in phylogenetic conflict, such as saturation in nucleotide changes, the genomic or morphological origin of the data, and variation in selective pressure on certain characters. Our analyses have shown that: (*i*) there is significant incongruence between phylogenies derived from morphological and molecular data, even when using matching taxa and algorithms; (*ii*) both morphological and molecular data show evidence of saturation; (*iii*) there are significant differences in tree topologies estimated from largely independently evolving genomes, even after accounting for systematic biases, such as base compositional bias superimposed on saturated sites; and (*iv*) alternative resolutions at key nodes are consistent with adaptive convergence in morphology arising from a shared ecological specialization (nectarivory), and might also arise in molecular data through shifts in selective pressure linked to gene function. We examined the evidence for each of these drivers of incongruence, and their relative contribution to phylogenetic conflict.

### (1) Methodological drivers of incongruence

#### (a) Taxonomic sampling

Incongruence between data types can arise through mislabeling samples and/or misidentifying taxa (Davis, Li & Murphy, 2010). With the rapid expansion of the GenBank database, and the push for compiling and analyzing large amounts of published data from diverse sources, there are fewer chances of replicating experiments that could help pinpoint errors in identification and labeling. Among phyllostomids, the published *Phyllostomus discolor RAG2* sequence is so similar to that of *Tonatia saurophila*, that it renders the genus *Phyllostomus* paraphyletic [see Electronic Supplementary Material Fig. 2 of Dumont *et al.* (2012)]. We found this error because each gene was analyzed separately and conflicting nodes were examined in depth. The conflict between the *RAG2* and *COX1* phylogenies—both data sets included exemplars of *Phyllostomus discolor* and *Tonatia saurophila*—stood out and the erroneous *RAG2* sequence could be removed. We also replicated DNA extraction and amplification of *RAG2* from vouchered specimens (L.M. Davalos & N.B. simmons, unpublished data), supporting our assessment of misidentification. Since the goal of Dumont *et al.* (2012) was to maximize taxon sampling, the incongruence was inadvertently missed and the error persisted. The documentation of sequences with their DNA source and museum vouchers (both are available for mislabeled or misidentified sequence GenBank accession FN641681), along with comparisons among gene trees can help reduce the frequency of this kind of error in supermatrices.

Previous morphological and molecular analyses of phyllostomids did not sample matching taxa, making direct comparisons of alternative trees difficult. If taxonomic sampling was the main source of incongruence between morphological and molecular hypotheses, then: (*i*) the original Wetterer *et al.* (2000) taxon sample should be significantly incongruent with the reference phylogeny; (*ii*) the old and new taxon samples of morphological data should yield significantly different phylogenies; and (*iii*) the new taxon sample used here should reduce conflict with the reference phylogeny.

To evaluate the first prediction, we filtered the posterior distribution of post-burn-in trees from analyses of the Baker *et al.* (2003) data set to estimate the posterior probability of the Wetterer *et al.* (2000) phylogeny. The morphology-based hypothesis was not found among the posterior trees (*P*≤ 0.00009). This conflict, however, could also arise from over-resolution of the molecular data set using Bayesian methods (Yang, 2007*a*). Therefore, we ran Bayesian analyses of the Wetterer *et al.* (2000) data set and evaluated the posterior probability of the Baker *et al.* (2000) phylogeny. Bayesian morphological analyses are not subject to the same overestimation bias as molecular data sets (Dávalos & Porzecanski, 2009), since the ratio of number of characters to tips is always much higher with molecular data (Yang, 2008). Significant conflict between phylogenies persisted, even with this conservative test (*P*≤ 0.00003).

We simultaneously sampled different taxa and added new characters to the morphological data set and had to separate effects of these two changes before testing the last two predictions. We ran a version of our data matrix without the 82 characters added since Wetterer *et al.* (2000). We then compared the resulting tree with the posterior distribution of trees from Bayesian analyses of the Wetterer *et al.* (2000) data set. Both phylogenies were obtained with matching characters, thus significant differences between them would result from differences in taxon sampling alone. Finding no significant differences would suggest that character sampling drives conflict between old and new morphological data sets. The phylogenies obtained with the new taxon sample were significantly different from those generated with the Wetterer *et al.* (2000) data (*P*≤ 0.00003), suggesting that taxon sample alone has a large effect. We also used the Wetterer *et al.* (2000) tree to filter the posterior distribution of Bayesian trees from the new data set. The new data significantly rejected the earlier morphological hypothesis (*P*≤ 0.000067). A similar analysis of the posterior distribution of trees from the Wetterer *et al.* (2000) data also uncovered significant incongruence between the two morphological phylogenies (*P*≤ 0.00003).

Likelihood-based tree comparisons were used to investigate if the new taxon sample reduced conflict with the reference phylogeny. The new morphological phylogeny was significantly unlikely given the Baker *et al.* (2003) data (*P*≤ 4E-04, Table 5). Individual partitions from that data set (mtrDNA and *RAG2*) also rejected the new morphological hypothesis (*P* < 1E–09, Table 5). Further, the new morphological data set significantly rejected the reference phylogeny (*P* < 0.000067). In short, given the Baker *et al.* (2003) data set (Table 5), both morphological phylogenies were significantly unlikely.

These analyses weakly support taxon sampling as a source of incongruence. The first two predictions were correct: the Wetterer *et al.* (2000) tree was significantly incongruent with the reference phylogeny, and the old and new taxon samples of morphological data yielded significantly different phylogenies, However, significant conflict between data types persisted and our third prediction was not met. Thus, taxon sampling may drive some, but certainly not all, the observed phylogenetic conflict.

#### (b) Number of characters sampled

Empirical data and simulation studies suggest that as increasingly large numbers of characters are sampled, the resulting hypothesis more closely resembles the underlying phylogeny, as long as statistically consistent models are used and the data are not systematically biased by ahistorical processes such as mutational bias leading to heterogeneity in base composition (Felsenstein, 1978; Sanderson *et al.*, 2000; Philippe, Lartillot & Brinkmann, 2005). Adding characters should reduce the conflict between morphological and molecular phylogenies. If sampling more characters reduced conflict then: (*i*) the new morphological data should reduce conflict with the reference phylogeny; and (*ii*) the molecular analyses presented here should reduce conflict with both old and new morphological data sets.

As explained above, the new morphological data did not reduce conflict with the Baker *et al.* (2003) data, despite additional taxonomic and character sampling. We examined the posterior probability of the new molecular trees in analyses of the original (Wetterer *et al.*, 2000) and new morphological data. The new molecular trees were rejected in every case (*P*≤ 0.00003), indicating no effect of expanded molecular sampling in reducing conflict. Analyses of the expanded molecular data did not reduce conflict with the morphological hypotheses. The complete molecular data set consistently rejected the morphological hypotheses (*P*≤ 6E-06 including saturated data, *P*≤ 1E–09 when excluding it), as did separate partitions (*P*≤ 4E–05, *cf*. Tables 5 and 6).

We also applied a node-by-node approach to comparing phylogenies. This approach is conservative because it breaks the phylogeny into parts, in contrast with whole-tree comparisons where a single node with very strong support can greatly change the likelihood of a phylogeny and result in a significant difference. Of the 49 ingroup nodes in the Wetterer *et al.* (2000) tree, 15 or 30% were shared with the reference phylogeny. The addition of 82 characters to the morphological matrix, and data from the *CYTB* and *COX1* mitochondrial genes to the molecular partition altered relationships among several groups within these partitions (*cf*. Figs 1 and 3). The trees, however, remained largely dissimilar, with the new morphology tree and the new molecular tree sharing only 20 of 57 (35%) potentially matching ingroup nodes (excluding taxa for which there were no molecular data).

The inclusion of new data increased statistical power to reject morphology-based phylogenies (Tables 5 and 6), and the percentage of nodes conflicting in morphological *versus* molecular analyses remained stable. We conclude that character sampling did not contribute substantially to the incongruence observed between morphological and molecular analyses for phyllostomids.

#### (c) Analytical methods

Prior studies of morphological data relied exclusively on MP for inferring phylogenies, in contrast with the Bayesian approach used in the reference phylogeny (Baker *et al.*, 2003). These alternative optimizations for inferring phylogeny are expected to result in different trees because of the sensitivity of model-based approaches to model selection (Buckley, Simon & Chambers, 2001; Yang & Rannala, 2005), and the overestimation of branch support in Bayesian analyses caused by the high ratio of characters to tips in molecular analyses (Alfaro, Zoller & Lutzoni, 2003; Yang, 2007*a*). If the method of analysis was the main methodological driver of phylogenetic incongruence, then: (*i*) morphology-based Bayesian phylogenies should reduce conflict with molecular phylogenies inferred using the same methods; and (*ii*) the MP morphological phylogeny should share more nodes with the MP molecular phylogeny than with the ML or Bayesian molecular phylogenies.

Bayesian analyses of both the Wetterer *et al.* (2000) data (*P*≤ 0.00003), and the new morphological data rejected all molecular phylogenies (*P*≤ 0.000067), suggesting that analytical method was not an important driver of conflict. A node-by-node examination of MP phylogenies revealed no decrease in conflict from applying the same methods to infer morphological and molecular phylogenies. Only 20 out of 57 possible common nodes (35%) were shared between MP morphological and molecular trees, and this proportion was no different from that shared by the morphology and ML molecular trees. Although there were more shared nodes in those trees than between the new morphology tree and the reference phylogeny (32% or 15 out of 46 possible nodes in common), the reduction in conflict from matching optimization algorithm was minimal. The impact of methods on congruence is too small to explain the observed conflict between morphological and molecular data.

### (2) Biological drivers of phylogenetic conflict

#### (a) Saturation in morphological changes

Previous morphological analyses failed to account for saturation in character states as a source of homoplasy that could contribute to conflict with molecular phylogenies. If saturation in morphological character states explained phylogenetic incongruence, then: (*i*) there will be fewer nodes conflicting with the molecular tree in the morphological phylogeny obtained from BYS analyses than from the MP analyses; and (*ii*) there will be fewer significant comparisons of the fit of the BYS morphological tree to the molecular data than with the MP tree. The results were consistent with the first prediction; only half the major conflicting nodes recovered in the MP analysis of the morphological data were recovered with BYS (Table 4). The conflicting nodes supported by morphology in both MP and BYS were mainly those supported by potentially convergent characters associated with feeding ecology. However, the BYS morphological tree was still not congruent with the molecular data (*P*≤ 6E-09, Table 5).

Applying a model of character evolution to a combination of true synapomorphies and homoplastic characters could help recover the underlying phylogeny, which would be obscured when using only MP (Lewis, 2001). The node-by-node analysis showed that both MP and the model-based approach recovered a small number of conflicting nodes (Table 4). Agreement with MP in recovering “incorrect” nodes, and significant incongruence between the Bayesian morphology tree and all molecular data sets imply that the model of morphological evolution did not fully account for the ahistorical signal in the morphological data. Both developmental and functional constraints limit the range of observed changes in morphology, and these limits may require more complex models than were implemented here. For example, the model of evolution applied to the morphological data assumed symmetrical rates of change from one state to another (Lewis, 2001), but a more complex model can implement asymmetrical rates from one state to another (Schultz & Churchill, 1999). Asymmetries in rates of change are expected when a character state can arise through more than one developmental pathway, resulting in inadequate assessments of homology between states (e.g. Bharathan *et al.*, 2002; Geeta *et al.*, 2012). Allowing asymmetrical rates of change for characters that arise through multiple pathways does not correct for the incorrect homology assessment, but accounts for the greater ease of change in one direction, providing a better fit of the model to the observations and a better estimate of phylogeny.

Another mechanism that results in multiple hits and saturation is adaptive convergence, when selective pressure on morphological function results in homoplasious character states. Across mammals, molecular phylogenies have helped identify convergent morphological changes associated with foraging ecology at multiple hierarchical levels from genera to orders (Hunter & Jernvall, 1995; Delsuc *et al.*, 2001; Reiss, 2001; Ruedi & Mayer, 2001). The Mkv model implemented here can potentially account for convergent changes, but only when the data include the single-taxon changes (or autapomorphies) that characterize long branches and can help identify adaptive convergent evolution (Lewis, 2001). Unfortunately, we collected only shared derived morphological characters, eschewing autapomorphies. This may explain why the BYS morphological phylogeny also recovered a number of conflicting nodes supported in MP. Despite being homoplasious, changes associated with dietary specialization could not be identified as such by the model. If this was the case, then the proportion of potentially convergent diet characters in support of conflicting nodes should be higher in nodes recovered with MP and BYS methods than those recovered only with MP. This prediction lacked statistical significance: the proportion of diet characters in support of conflicting nodes recovered with MP and BYS was 0.70, and in nodes recovered only with MP was 0.33 (unpaired one-sided t-test *P* = 0.11, see Table 4). To take advantage of currently available models of evolution an approach to morphological data collection that includes autapomorphies may be necessary to minimize the consequences of saturation in morphological data sets.

Based on extensive experience with saturation in molecular data, we anticipate that analyzing morphological data in a model-based framework requires: (*i*) excluding fast-evolving characters; (*ii*) identifying functional categories of characters and accounting for their properties (e.g. rates of change, change asymmetry); (*iii*) using partitioned models; and (*iv*) modeling site heterogeneity for functional partitions of the data (Lartillot, Brinkmann & Philippe, 2007; Rodríguez-Ezpeleta *et al.*, 2007; Dávalos & Perkins, 2008; Wagner, 2012).

#### (b) Widespread homoplasy in morphological data

We analyzed the possibility that homoplasy in morphological data caused the conflict with molecular phylogenies. Generalized homoplasy in morphological characters has been found in comparison with molecular data designed to test mammalian inter-ordinal relationships (Springer *et al.*, 2007). If this were the case with our phyllostomid data, the morphological data optimized on the reference phylogeny would result in few homologous changes, especially relative to other molecular data. Instead, we found that homologous changes were disproportionately more common in morphological data than among mitochondrial characters (Fig. 9), and even the original Baker *et al.* (2003) data (Fig. 10B). Further, perfectly homologous optimizations to the reference phylogeny were not confined to any one particular kind of morphological data, but were distributed across the six classes we identified above (pelage and integument, skull and dentition, postcranium, hyoid apparatus, tongue, and internal). These analyses are consistent with homoplasy being localized to subsets, but not entire classes of characters (e.g. to those directly associated with adaptive convergence in feeding ecology, but not to all tongue characters).

#### (c) Saturation in molecular substitutions

To date, analyses of molecular data to resolve relationships across Phyllostomidae have not applied models to account explicitly for saturation in substitutions, particularly at silent third codon positions (e.g. Baker *et al.*, 2003, 2000; Datzmann *et al.*, 2010). We uncovered extensive saturation in mitochondrial sequences particularly at third positions and in 16S loops (Table 2), and these sites also differed significantly in base composition. If biases in base composition superimposed on sites where substitutions were saturated produced phylogenetic conflict, then: (*i*) support for nodes in conflict would decline or disappear after down-weighting or excluding those sites; (*ii*) support for conflicting nodes will concentrate at mt third codon positions and mtr loops; and (*iii*) analyses down-weighting or excluding these data will result in fewer significant conflicts between molecular partitions and individual trees.

There is some evidence for the first prediction: support for the position of *Lonchorhina* declined after excluding saturated sites and down-weighting semi-saturated sites (Table 4). There was significant support from mt third codon positions and loops for Micronycterinae being the sister taxon of the remaining phyllostomids (Fig. 11A). However, the origin of support for that node changed when analyses fitted separate models to each partition (Fig. 11B). *RAG2* third positions, which were neither saturated nor different in base composition, consistently supported that node. PLS analyses showed that saturated sites did not consistently support Micronycterinae as the sister taxon of the remaining phyllostomids, the position of *Lonchorhina*, or the monophyly of nectar feeders (Fig. 11). Saturation did generate conflict between the mitochondrial data and *RAG2* sequences, with conflict going from being significant (*P* = 0.008) to non-significant (*P*≥ 0.547, *cf*. Tables 5 and 6).

Down-weighting or excluding saturated sites resulted in lower measures of support in most phylogenies (*cf*. Figs 5 and 6). The low slopes (0.00–0.02) of transitions at mitochondrial third codon positions and 16S loops indicated these were accumulating changes independently from shared evolutionary history (Table 2). By definition their contribution to phylogenetic resolution is noise. Transversions at those sites were not as saturated (aside from third positions of *COX1*, slopes ranged from 0.24 to 0.43), indicating that in some parts of the phylogeny these sites contributed some signal, and not only noise.

Accounting for the effects of saturation and base compositional bias reduced conflict across the phylogenies, even if saturation did not account for the resolution of key nodes as outlined above. Substitutional saturation is an important source of incongruence in data available for phyllostomids, and has long been recognized as a source of conflict in general (Phillips *et al.*, 2004; Gibson *et al.*, 2005; Baurain, Brinkmann & Philippe, 2007; Dávalos & Perkins, 2008).

#### (d) Incongruence between gene trees

Conflict between mitochondrial and nuclear phylogenies among phyllostomids has been highlighted before (Velazco & Patterson, 2008), and has been implied in the different resolutions obtained by results of separate analyses of mt and nuclear data (Baker *et al.*, 2003, 2000). If different gene histories explained phylogenetic conflict between molecular data sets, then: (*i*) there should be significant incongruence between different genes; and (*ii*) conflict will persist even after correcting for systematic biases such as base compositional heterogeneity superimposed on mutational saturation. We uncovered significant incongruence between *RAG2* and the mtrDNA data, despite using a partitioned model to reduce the impact of saturation on the phylogeny (*P*≤ 0.044, Table 5). Down-weighting saturated sites when estimating the mtrDNA phylogeny did not render the conflict non-significant (*P*≥ 0.009, Table 6). Our results are consistent with different gene trees underlying the phylogenetic conflict between mt and nuclear data.

Several processes can generate incongruence between gene trees, particularly when these genes correspond to genomes with distinct modes of inheritance, as is the case here. These processes include: (*i*) paralogy (Fawcett, Maere & Van de Peer, 2009); (*ii*) lateral gene transfer (Bapteste *et al.*, 2005; Hotopp *et al.*, 2007); (*iii*) introgression (Hailer & Leonard, 2008); (*iv*) incomplete lineage sorting (Pollard *et al.*, 2006; Heckman *et al.*, 2007); (*v*) poor taxon sampling and outgroup choice (Hedtke, Townsend & Hillis, 2006; de la Torre-Bárcena *et al.*, 2009); and (*vi*) adaptive convergence (Li *et al.*, 2008, 2010; Liu *et al.*, 2010).

Paralogy could affect either the nuclear or mt sequences, so that gene copies from some individuals are not orthologous to those of others and, having a different gene history, result in significantly incongruent trees. The structure, expression, and function of the recombination activating genes *RAG1* and *2* have been studied in depth in humans (Oettinger *et al.*, 1990; Corneo *et al.*, 2001; Sadofsky, 2004), and paralogy with other genes has not been reported. In bats, both *RAG1* and *RAG2* have been used as phylogenetic markers, and their sequences, although paralogous, are distinct and do not co-amplify (Teeling *et al.*, 2003, 2005). Alternatively, paralogy could affect the mitochondrial data through co-amplification of insertions of mitochondrial sequences onto the host nucleus (Bensasson, Feldman & Petrov, 2003; Anthony *et al.*, 2007). These insertion events are relatively common within mammals (e.g. Olson & Yoder, 2002; Antunes *et al.*, 2007; Hazkani-Covo & Graur, 2007), and could mislead phylogenetic analyses because the nuclear sequence (or Numt, for nuclear-mt insertion) changes more slowly and is inherited biparentally, unlike the orthologous copy of the gene in the mitochondrial genome. We searched for frame-shifts and stop codons in the concatenated *CYTB* and *COX1* alignment to detect the presence of Numts in our data, but found no indication of such insertions. We conducted similar comparisons using the structural mtr alignment, and found no evidence of Numts [although these might not always be detectable through structural analysis, see Olson & Yoder (2002)]. There is currently no support for paralogy underlying incongruence between mt and nuclear genes with these data.

Although frequently invoked in prokaryote evolution, lateral gene transfer (LGT) can also affect multicellular eukaryotes, e.g. through the transfer of genes from bacterial endosymbionts to the host (Hotopp *et al.*, 2007). This kind of LGT is an unlikely explanation for the observed incongruence because bacterial endosymbionts have not been reported in mammals; and gene loss in other genomes is the more likely explanation for genes thought to have transferred from prokaryotes to eukaryotes (Salzberg *et al.*, 2001). Most cases of LGT reported in prokaryotes are among genes present in more than one copy (Lerat, Daubin & Moran, 2003), or mediated by transposable genetic elements called “transposons” (Xu *et al.*, 2007). The nuclear marker studied here (*RAG2*) could fit the profile of laterally transferred genes because it has a paralogue—*RAG1*—and therefore is present in more than one copy (Sadofsky, 2004). Further, the function and structure of the genes are consistent with an origin by insertion of a transposon or “jumping gene” (Market & Papavasiliou, 2003), and recombination activating genes appear abruptly in evolution beginning with jawed vertebrates (Schluter *et al.*, 1999). The proposed transfer event into ancestral vertebrates, however, would not explain phylogenetic conflict within phyllostomids unless more recent transposon activity was found. Since the conflict we document here is with the virtually independently evolving mitochondrial genome, comparisons with other nuclear genes are necessary to identify a pattern consistent with more recent gene transfer.

A pattern of phylogenetic conflict between *RAG2* and a mitochondrial gene is also consistent with introgression and incomplete lineage sorting, and these have been documented in vertebrates much more commonly than LGT. Introgression has been used to explain conflict between mitochondrial haplotype diversity and coalescent pattern and either morphologically delineated evolutionary units, or phylogenies based on nuclear data (e.g. Dávalos, 2005; Russell *et al.*, 2008). More extensive analyses with dense population samples from more than two independent loci have uncovered patterns of genetic diversity and differentiation consistent with female- (Berthier, Excoffier & Ruedi, 2006), or male-mediated introgression (Mao *et al.*, 2010). Although previous analyses have all suggested or uncovered introgression among close relatives, the long-term phylogenetic signature of those events would be incongruence between genomes inherited maternally, paternally, and/or bi-parentally. Sampling more nuclear markers could help test introgression as an explanation for phylogenetic conflict: there should be congruence across nuclear phylogenies and conflict with the mt phylogeny if sex-specific introgression explains the conflict. More complicated scenarios, such as recurrent hybridization leading to genetic admixture in an important fraction of the population (25%) of one species (Berthier *et al.*, 2006), should produce more complex patterns of incongruence among nuclear genes, and would require dense sampling of the genome to uncover after a series of speciation events (Eckert & Carstens, 2008).

In contrast with a simple sex-specific introgression scenario, lineage sorting of ancestral polymorphism is expected to generate incongruence between phylogenies from genes in the same genome (Degnan & Rosenberg, 2006). The development of new approaches to infer species phylogeny while accounting for conflicting gene trees arising from independent coalescent processes (reviewed by Liu *et al.*, 2009), has renewed interest in accounting for lineage sorting. New methods seek to model the coalescent process and therefore estimate some of the population-level parameters that give rise to lineage sorting in the first place (e.g. ancestral molecular diversity, effective population size). Multiple alleles per species from several loci are therefore needed to parameterize such models, significantly increasing data requirements (e.g. Brumfield *et al.*, 2008; Liu *et al.*, 2008). Determining if lineage sorting and/or introgression are driving the conflict observed requires collecting multi-allele, multi-locus nuclear data that are currently unavailable for this system.

Sampling more taxa to break up long branches and reduce systematic bias can improve estimates of phylogeny (Graybeal, 1998; Zwickl & Hillis, 2002), and resolve significant conflict among gene trees (Hedtke *et al.*, 2006). Further, using a single distantly related outgroup to polarize characters results in incongruence if it makes the root of each gene tree random (de la Torre-Bárcena *et al.*, 2009). Incongruence among gene trees in mammals, and bats in particular, has been traced to differing taxon samples and poor choices in rooting phylogenies (Van Den Bussche & Hoofer, 2004). Our taxon sample aimed to include the diversity of the family and, although individual genes were not available for the entire sample, tests of incongruence encompassed at least 45 species representing the taxonomic diversity of the family. The choice of outgroups (see Section II.1*a*) encompassed a suite of nine species from both closely (i.e. in the superfamily Noctlionoidea) and more distantly related taxa. If phylogenetic conflict was the result of poor taxonomic sampling and/or a poor choice of outgroup, expanding taxon and outgroup sampling would reduce the conflict between gene trees. Instead, incongruence persisted despite adding five more species, two of them outgroups (*cf*. Tables 5 and 6). These results indicate that taxon and outgroup sampling are probably not driving the conflict.

Paralogy, lateral gene transfer, and taxon/outgroup sampling can be ruled out as drivers of phylogenetic conflict between gene trees with our data. Evaluating the roles of lineage sorting of ancestral polymorphism and introgression in generating conflict will require collecting data from many more individuals from each species and across more genes than were analyzed here.

#### (e) Adaptive convergence in morphology

Convergent evolution resulting from ecological adaptation can generate homoplasy that manifests as conflict between the phylogeny generated using convergent characters and other trees (Gatesy *et al.*, 1996; Delsuc *et al.*, 2001; Wiens *et al.*, 2003). If convergent evolution linked to feeding ecology resulted in phylogenetic conflict, then: (*i*) potentially spurious clades would be supported mainly by characters related to feeding ecology; and (*ii*) excluding those characters from analyses should break up the potentially spurious clade. Characters linked to feeding ecology made up 50% or more of the unambiguous transformations supporting four of the six nodes examined (Table 4). Excluding potentially convergent characters broke up the clade comprising the nectar-feeding subfamilies Glossophaginae and Lonchophyllinae.

We propose that the clade uniting the nectar-feeding bats is the result of morphological similarities associated with ecological adaptations to nectar feeding. This conclusion is based on the dietary specialization of the clade, the identity of the synapomorphies supporting it, the loss of the node after excluding the characters associated with feeding ecology using both MP and BYS algorithms (Table 4), and the extensive homoplasy seen in those characters when mapped onto the reference phylogeny. Questions regarding the monophyly of nectar-feeding phyllostomids arose as early as the 1960s (reviewed by Griffiths, 1982). Close examination of the muscle and tongue morphology led Griffiths (1982, 1983) to propose that Lonchophyllinae evolved nectarivory independently from Glossophaginae and that these taxa were not sister taxa. Both Wetterer *et al.*'s (2000) analyses and the ones presented here recovered monophyletic nectar-feeding clades that excluded the genus *Brachyphylla* (Figs 1 and 3). These morphological results directly contradict some prior morphological studies and all relevant molecular analyses (Griffiths, 1982, 1983; Baker *et al.*, 2003, 2000; Datzmann *et al.*, 2010).

#### (f) Potentially adaptive convergent molecular evolution

Just as morphological features may converge on a similar phenotype under selective pressure associated with ecological specialization, genes facing selection for a particular function may also converge. For example, convergence in the protein sequences of the *Prestin* gene, associated with sensitivity and frequency selectivity of the cochlea in mammals, has been proposed to explain spurious clades found in trees based on protein sequences in bats and among cetaceans (Li *et al.*, 2008, 2010; Liu *et al.*, 2010). Based on the results of analyses of the concatenated molecular data, we examined the node uniting the nectar-feeding Lonchophyllinae and Glossophaginae (Table 4, Fig. 5). If adaptive convergence caused the conflict between the molecular resolution obtained here and earlier molecular phylogenies (*cf*. Figs 2 and 5), then: (*i*) support for this node should come from functional parts of the genes involved; (*ii*) selection should be operating on the genes at rates different from those of other lineages; (*iii*) and there should be a link between the gene and ecological function. We found that—as in studies of snakes and agamid lizards (Castoe *et al.*, 2009)—support for the spurious nectar-feeding clade using molecular data stemmed from substitutions that result in amino acid changes in mitochondrial protein-coding genes (Fig. 11A). This is also consistent with the fact that including the mitochondrial protein-coding data is the main difference in gene sampling between the data analyzed here and those of Baker *et al.* (2003).

It is more difficult to implicate selection in producing the spurious clade. The shift in selection pressure detected at 18 amino acids in the *CYTB* gene across the two nectar-feeding lineages was linked to both high support and high rejection of the spurious clade, rather than just to high support. The amino acids under shifting selection comprise a proportion of shared changes in nectar-feeding phyllostomids, but also a smaller proportion of changes distinctive to each branch, or autapomorphies of these two subfamilies (Fig. 12B). Half the amino acid changes (eight of 16) that provided significant support to the spurious clade correspond to regions of *CYTB* and two amino acids in *COX1* experiencing background selection (Fig. 12A). Finally, the shift in selection detected can be interpreted as relaxation of purifying selection (e.g. Zhao *et al.*, 2010), or as the result of a brief burst of positive selection in the nectar-feeding lineages (e.g. Li *et al.*, 2007) (Table 7). Relaxed selection is more parsimoniously explained by genetic drift resulting from small population size (e.g. Moran, 1996; Ingman & Gyllensten, 2007), rather than by changes in selection pressure. Demographic changes are expected to leave signatures across the entire genome (Russell *et al.*, 2011), and examining *K*_*a*_*/K*_*s*_ across many loci can help test this hypothesis. The critical importance of the cytochrome *b* protein in all eukaryotes (discussed below), the conservation of its function across mitochondria, chloroplasts, and cyanobacteria, and the non-random distribution of sites under shifting selection relative to the structure of the protein (Fig. 13), weakly support the hypothesis of a brief burst of positive selection. Change in *K*_*a*_*/K*_*s*_ in the evolutionary history of these lineages is consistent with positive selection toward a particular genotype, but it cannot explain all the phylogenetic support for the spurious clade. Selection as a source of support for the spurious clade is a hypothesis that merits further study.

The link between the *CYTB* gene and function is clear, but not exclusive to the particular ecology of nectar-feeding phyllostomids. The cytochrome *b* protein is a component of the respiratory complex involved in electron transport across the mitochondrial membrane, which generates an electrochemical potential indispensable in ATP synthesis (Esposti *et al.*, 1993). Specifically, cytochrome *b* is a respiratory subunit that forms centres at each side of the mitochondrial membrane that react with the coenzyme ubiquinone and results in oxidation/reduction of the ferricytochrome complex and trans-membrane electron transfer (Mitchell, 1975; Iwata *et al.*, 1998; Cramer *et al.*, 2006). In humans, mutations in *CYTB* can lead to sudden infant death, cardiac defects, neurological defects, deafness, epilepsy, growth retardation, mental retardation, and muscle weakness (Keightley *et al.*, 2000). Although the function of this enzyme has not been documented in bats, its centrality to respiration and energy production in the cell, and the range of deleterious consequences of mutation in humans imply that the cytochrome *b* protein is of critical importance to fitness. The sites under shifting selection were significantly associated with two broad regions: trans-membrane helices, responsible for trans-membrane electron transfer (Trumpower & Gennis, 1994), and the carboxy-terminal domain of the protein, essential for correct assembly of the entire respiratory complex (di Rago *et al.*, 1993) (Fig. 13).

Based on what is known about the protein's function from model organisms, we speculate that these sites might be of particular importance to nectar-feeding bats because of their high energy requirements. Phyllostomid nectar-feeders have extremely high metabolic rates (Kelm *et al.*, 2011), which in turn result in extreme metabolic adaptations such as near-maximum rates of nutrient absorption (Winter, 1998), and almost exclusive use of dietary carbohydrates in respiration, instead of fat reserves (Voigt & Speakman, 2007). At present, the relationship between amino acid replacements in the trans-membrane helices and carboxy-terminus of *CYTB* in nectar-feeding phyllostomids is still a conjecture that can be tested with studies coupling whole-organism performance to allelic variants and their *in vitro* biochemical activity, and corroborated by examining *CYTB* variation among other “high-energy” lineages descended from generalized plant-visiting ancestors.

The first two predictions concerning the impact of adaptive convergence on incongruence were met: amino acid substitutions in functional parts of the *CYTB* gene (trans-membrane helices and carboxy-terminus) support the potentially spurious node, and these replacements may be the result of a short burst of positive selection. There is also a potential link between the gene and ecological function and a potential mechanism generating convergence: selection as a result of demand for high-performance energy metabolism in these lineages. The link between support for the clade and molecular selection is ambiguous, and the connection between bat ecology and protein function is inferential. For these reasons we consider the adaptive convergence hypothesis weakly supported, but worthy of further research.

## V. CONCLUSIONS

1. The persistence of significant phylogenetic incongruence even after controlling for molecular saturation, and the limitations of current analyses when morphological data are collected without the expectation of saturation, cautions against the impulse to uncritically adopt hypotheses based on data combination. Instead, the approaches used here point to the data necessary to estimate a phylogeny with minimal systematic bias: multi-locus multi-allele molecular data, and the application of models of character evolution capable of accounting for saturation, including collecting morphological data from autapomorphic characters and states. Despite accounting for more than a third of all chiropteran phylogenies (Jones *et al.*, 2002), key aspects of phyllostomid phylogeny remain uncertain.
2. Significant prior work on incongruence has focused on the morphology *versus* molecules debate. In the vast majority of cases those conflicts are between traditional taxonomy or systematics and MP morphological phylogenies, and new molecular ML or Bayesian phylogenies. Either way, the conflict may arise purely for methodological and not biological reasons. The data collected and analyzed here indicate that phylogenetic conflict arose chiefly from biological processes that shape organisms and clades. Insofar as these biological processes are thought to be common, phylogenetic conflict will constitute a feature of phylogeny estimation. No special type of data can guarantee the lack of systematic bias arising from biological processes, indeed morphological data were better than average molecular characters in recovering homologous changes. To overcome phylogenetic conflict, biologists will need to understand the processes that bias all data types, and this need will only grow as phylogenies move from single-system or gene to multiple systems and loci.
3. Morphological data can become saturated and biased. Complex biological processes underlie saturation and uncritically applying available models will not be enough to correct the resulting biases. Estimating “total evidence” phylogenies using morphological and molecular data will require improvements both in data collection and exclusion, and improvements to evolutionary models. This complex task will become more successful when data are collected to allow models of evolution to account for the resulting biases, not because data can be made to be completely free of systematic biases.
4. Investigating the range of biological processes underlying phylogenetic conflict is informative in its own right, not just for the sake of estimating phylogeny. These analyses illuminate the processes shaping organisms through evolutionary history, and provide starting points to examine questions such as the rates of change in different molecular and morphological characters and their possible relationship to demographic changes or shifts in the strength of natural selection. By identifying the taxa and nodes where conflict concentrates, these analyses can also help guide efficient future data collection.
5. Adaptive convergence in gene sequences resulting from adaptation to particular ecological function is a potential source of phylogenetic conflict in phyllostomids. The hypothesis of molecular convergence in mitochondrial *CYTB* to explain phylogenetic support for a nectar-feeding clade is weakly supported, but has the potential to connect phylogeny to physiological adaptations to meet high-energy demands in two clades of nectar-feeding bats in this family.

## VI. ACKNOWLEDGEMENTS

For access to specimens throughout the course of this study we thank M. Carleton, the late C. Handley, L. Gordon (USNM), B. Patterson (FMNH), and P. Myers (UMMZ). G. Dent (AMNH) provided invaluable assistance in the use of the Museum's computer cluster. A. Corthals and P. Velazco generously read and commented on early drafts of this manuscript, and we also thank two anonymous reviewers for detailed and extremely helpful reviews. Financial support for A.L.C. was provided by the Center for Biodiversity and Conservation and the Office of Grants and Fellowships of the AMNH, and Columbia University's Center for Environmental Research and Conservation. Some bioinformatics analyses were carried out on the freely available Oslo Bioportal (www.bioportal.uio.no), others on the AMNH's Science Computer Cluster Facility, and another part of this work was carried out using the resources of the Computational Biology Service Unit at Cornell University, which is partially funded by Microsoft Corporation. This research was supported in part by the NSF through DEB-0949759 to L.M.D., and DEB-0949859 to N.B.S.

## Supporting Information

Appendix S1. Genbank accessions of DNA sequences analysed.
