# Supplementary material for: Understanding phylogenetic incongruence: lessons from phyllostomid bats
Source: Biol Rev Camb Philos Soc. 2012 Aug 14;87(4):991–1024. doi: 10.1111/j.1469-185X.2012.00240.x (PMC3573643; doi:10.1111/j.1469-185X.2012.00240.x)
Supplement: Appendix S1. — Genbank accessions of DNA sequences analysed. [file brv0087-0991-sd1.doc]

Appendix S1. GenBank accessions of DNA sequences analysed.

| **Species** | **mtrDNA** | ***COX1*** | ***CYTB*** | ***RAG2*** |
| --- | --- | --- | --- | --- |
| ***Ametrida centurio*** | AY395802.1 | EF079971.1 | AY604446.1 | AF316430.1 |
| ***Anoura caudifer*** | AY395835.1 | EF079981.1 | L19506.1 |  |
| ***Anoura geoffroyi*** |  | EF079996.1 |  | AF316431.1 |
| ***Ardops nichollsi*** | AY395803.1 |  | AY572337.1 | AF316434.1 |
| ***Ariteus flavescens*** | AY395804.1 |  | AY604436.1 | AF316435.1 |
| ***Artibeus cinereus*** | AY395810.1 | EF080047.1 | U66511.1 | AF316443.1 |
| ***Artibeus concolor*** |  | JF452412.1 | U66519.1 | AF316432.1 |
| ***Artibeus hirsutus*** |  |  | AY684777.1 | AF316433.1 |
| ***Artibeus jamaicensis*** | AF061340.1 | AF061340.1 | GQ861667.1 | AY834663.1 |
| ***Brachyphylla cavernarum*** | AY395806.1 |  | AY620467.1 | AF316436.1 |
| ***Carollia brevicauda*** |  | JF453684.1 | AF511951.1 | AF316437.1 |
| ***Carollia perspicillata*** | AY395836.1 | EF080211.1 | AF511991.1 |  |
| ***Centurio senex*** | AF263227.1 |  | AY604444.1 | AF316438.1 |
| ***Chiroderma villosum*** |  | EF080289.1 | DQ312414.1 | AF316439.1 |
| ***Choeroniscus godmani*** |  | EU096698.1 |  | AF316440.1 |
| ***Choeronycteris mexicana*** | AY395808.1 |  |  | AF316441.1 |
| ***Chrotopterus auritus*** | AF411538.1 | EF080303.1 |  | AF316442.1 |
| ***Desmodus rotundus*** | AF263228.1 | JF435307.1 | DQ077398.1 | AF316444.1 |
| ***Diaemus youngi*** | AF411534.1 | EF080328.1 |  | AF316445.1 |
| ***Diphylla ecaudata*** | AF411533.1 |  | DQ077399.1 | AF316447.1 |
| ***Ectophylla alba*** | AY395811.1 |  | DQ312404.1 | AF316448.1 |
| ***Enchisthenes hartii*** | AY395838.1 | EU161064.1 | U66517.1 | AF316449.1 |
| ***Erophylla sezekorni*** | AY395839.1 |  | GU937254.1 | AF316450.1 |
| ***Furipterus horrens*** | AF345922.1 | EU096742.1 | AY621004.1 | AY141016.1 |
| ***Glossophaga longirostris*** |  | JF454671.1 | AF382875.1 |  |
| ***Glossophaga soricina*** | AY395840.1 | EF080360.1 | AF423081.1 | AF316452.1 |
| ***Glyphonycteris daviesi*** | AY395812.1 | EF080364.1 | AY380747.1 | AF316464.1 |
| ***Glyphonycteris sylvestris*** | AY395841.1 | EF080366.1 | AY380746.1 | AF316471.1 |
| ***Hylonycteris underwoodi*** | AY395813.1 |  |  | AF316453.1 |
| ***Lampronycteris brachyotis*** | AF411536.1 | EF080370.1 | AY380748.1 | AF316463.1 |
| ***Leptonycteris yerbabuenae*** | AY395814.1 |  | AF382889.1 | AF316454.1 |
| ***Lionycteris spurrelli*** | AY395815.1 | EF080374.1 | AF423100.1 | AF316455.1 |
| ***Lonchophylla robusta*** |  |  | AF423091.1 |  |
| ***Lonchophylla thomasi*** | AY395842.1 | EF080377.1 | AF423086.1 | AF316456.1 |
| ***Lonchorhina aurita*** | AY395843.1 | JF447260.1 | FJ155494.1 | AF316457.1 |
| ***Lophostoma brasiliense*** | AF411544.1 | EF080416.1 |  | AF316489.1 |
| ***Lophostoma silvicolum*** | AF263230.1 | EF080433.1 | DQ903830.1 | AF442082.1 |
| ***Macrophyllum macrophyllum*** | AF411540.1 | EU096773.1 | FJ155484.1 | AF316458.1 |
| ***Macrotus californicus*** |  |  | AY380744.1 | AF316459.1 |
| ***Macrotus waterhousii*** | AF263229.1 |  | AY380745.1 | AF316461.1 |
| ***Mesophylla macconnelli*** | AY395818.1 | EF080443.1 | AY157042.1 | AF316462.1 |
| ***Metavampyressa nymphaea*** |  |  | DQ312418.1 |  |
| ***Micronycteris hirsuta*** | AY395819.1 | EF080447.1 | AY380769.1 | AF316465.1 |
| ***Micronycteris megalotis*** | AY395821.1 | EU096780.1 | DQ077426.1 | AF316467.1 |
| ***Micronycteris minuta*** | AY395823.1 |  | AY380752.1 | AF316468.1 |
| ***Mimon bennettii*** |  |  | DQ903832.1 |  |
| ***Mimon crenulatum*** | AF411543.1 | EU096781.1 | FJ155478.1 | AF316472.1 |
| ***Monophyllus redmani*** |  |  | AF382888.1 | AF316473.1 |
| ***Mormoops megalophylla*** | AF407174.1 |  | AF330808.1 | AF338702.1 |
| ***Musonycteris harrisoni*** | AY395844.1 |  |  | AF316475.1 |
| ***Mystacina tuberculata*** | AF263222.1 | AY960981.1 | AF144068.1 | AY141021.1 |
| ***Noctilio leporinus*** | AF263224.1 | EF080534.1 | AF330796.1 | AF330816.1 |
| ***Phylloderma stenops*** | AF411542.1 | EU096830.1 | FJ155480.1 | AF316480.1 |
| ***Phyllonycteris aphylla*** |  |  | AF187033.1 | AF316478.1 |
| ***Phyllops falcatus*** |  |  | DQ211651.1 | AY604453.1 |
| ***Phyllostomus discolor*** |  | EF080546.1 |  |  |
| ***Phyllostomus hastatus*** | AF411541.1 | JF455375.1 | FJ155479.1 | AF316479.1 |
| ***Platalina genovensium*** |  |  | AF423101.1 |  |
| ***Platyrrhinus helleri*** |  | EF080579.1 | FJ154140.1 | AF316481.1 |
| ***Pteronotus davyi*** | AF407176.1 |  | AF338671.1 | AF338692.1 |
| ***Pteronotus parnellii*** |  | EU096905.1 | AF338661.1 | AF330817.1 |
| ***Pygoderma bilabiatum*** | AY395826.1 |  | AY604438.1 | AF316483.1 |
| ***Rhinophylla pumilio*** | AY395827.1 | EF080598.1 | AF187031.1 | AF316484.1 |
| ***Saccopteryx bilineata*** | AF263213.1 | EF080678.1 | EF584202.1 | AY141015.1 |
| ***Sphaeronycteris toxophyllum*** | AY395828.1 |  | AY604451.1 | AF316486.1 |
| ***Stenoderma rufum*** | AY395829.1 |  | AY604432.1 | AF316487.1 |
| ***Sturnira lilium*** |  | EF080684.1 | DQ312398.1 | AF316488.1 |
| ***Thyroptera tricolor*** | AF263233.1 | EU097048.1 | AY621005.1 | AY141028.1 |
| ***Tonatia saurophila*** | AF411530.1 | EF080734.1 |  | AF442086.1 |
| ***Trachops cirrhosus*** | AF411539.1 | EF080747.1 | DQ233669.1 | AF316490.1 |
| ***Trinycteris nicefori*** | AY395830.1 | EF080784.1 | AY380749.1 | AF316469.1 |
| ***Uroderma bilobatum*** | AY395831.1 | EF080788.1 | AY169955.1 | AF316491.1 |
| ***Vampyressa pusilla*** | AY395832.1 |  | AY157054.1 | AF316493.1 |
| ***Vampyriscus bidens*** | AY395833.1 | EF080792.1 | AY157055.1 | AF316492.1 |
| ***Vampyrodes caraccioli*** | AY395846.1 | EF080804.1 | AY157034.1 | AF316494.1 |
| ***Vampyrum spectrum*** |  | EF080809.1 | FJ155482.1 | AF316495.1 |
